# Transcriptome analysis of two *Pogostemon cablin* chemotypes reveals genes related to patchouli alcohol biosynthesis

**DOI:** 10.7717/peerj.12025

**Published:** 2021-08-26

**Authors:** Wuping Yan, Zhouchen Ye, Shijia Cao, Guanglong Yao, Jing Yu, Dongmei Yang, Ping Chen, Junfeng Zhang, Yougen Wu

**Affiliations:** College of Horticulture, Hainan University, Haikou, Hainan, China

**Keywords:** Different chemotypes, Differentially expressed genes, Patchouli alcohol biosynthesis, *Pogostemon cablin*, Transcriptome, Transcription factors

## Abstract

*Pogostemon cablin*, a medicinally and economically important perennial herb, is cultivated around the world due to its medicinal and aromatic properties. Different *P. cablin* cultivars exhibit different morphological traits and patchouli oil components and contents (especially patchouli alcohol (PA) and pogostone (PO)). According to the signature constituent of the leaf, *P. cablin* was classified into two different chemotypes, including PA-type and PO-type. To better understand the molecular mechanisms of PA biosynthesis, the transcriptomes of Chinese-cultivated *P. cablin* cv. PA-type “Nanxiang” (NX) and PO-type “Paixiang” (PX) were analyzed and compared with ribonucleic acid sequencing (RNA-Seq) technology. We obtained a total of 36.83 G clean bases from the two chemotypes, compared them with seven databases and revealed 45,394 annotated unigenes. Thirty-six candidate unigenes participating in the biosynthesis of PA were found in the *P. cablin* transcriptomes. Overall, 8,390 differentially expressed unigenes were identified between the chemotypes, including 2,467 upregulated and 5,923 downregulated unigenes. Furthermore, six and nine differentially expressed genes (DEGs) were mapped to the terpenoid backbone biosynthetic and sesquiterpenoid and triterpenoid biosynthetic pathways, respectively. One key sesquiterpene synthase gene involved in the sesquiterpenoid and triterpenoid biosynthetic pathways, encoding patchoulol synthase variant 1, was significantly upregulated in NX. Additionally, GC-MS analysis of the two chemotypes in this study showed that the content of PA in NX was significantly higher than that of PX, while the content of PO showed the opposite phenotype. Quantitative real-time polymerase chain reaction (qRT-PCR) analysis showed that the DEG expression tendency was consistent with the transcriptome sequencing results. Overall, 23 AP2/ERF, 13 bHLH, 11 MYB, 11 NAC, three Trihelix, 10 WRKY and three bZIP genes that were differentially expressed may act as regulators of terpenoid biosynthesis. Altogether, 8,314 SSRs were recognized within 6,825 unigenes, with a distribution frequency of 18.32%, among which 1,202 unigenes contained more than one SSR. The transcriptomic characteristics of the two *P. cablin* chemotypes are comprehensively reported in this study, and these results will contribute to a better understanding of the molecular mechanism of PA biosynthesis. Our transcriptome data also provide a valuable genetic resource for further studies on *P. cablin*.

## Introduction

*Pogostemon cablin* (Blanco) Benth. is a perennial herb belonging to Labiatae (Lamiaceae). It is recorded in the Chinese Pharmacopeia (China Pharmacopoeia Committee, 2015) due to its medicinal and economic importance. It is native to southern and south-eastern Asia (Maheshwari et al., 1993). Its stem and leaves, which are commonly known as “Guanghuoxiang”, can be used to extract patchouli oil and have been used in traditional medicine in China to treat indigestion, gastroenteritis, headache, fever and so on. In addition, patchouli oil contains abundant patchouli alcohol (PA, also known as patchoulol) and pogostone (PO, also known as dhelwangin) and is an important ingredient used as a base that provides lasting character to fragrances in the perfume industry (Zhang et al., 2018). *P. cablin* was brought to China for perfume and medicinal uses as early as or potentially before the Liang Dynasty (Wu, Guo & Zheng, 2007). Currently, *P. cablin* is widespread in southern China, and Shipai, Zhanjiang, and Gaoyao in Guangdong Province along with Wanning and Haikou in Hainan Province are the main *P. cablin*-growing regions. *P. cablin* is separated into *P. cablin* cv. “Nanxiang” (NX, grown in Hainan), *P. cablin* cv. “Paixiang” (PX, grown in Guangzhou), *P. cablin* cv. “Zhanxiang” (ZNX, grown in Zhanjiang) and *P. cablin* cv. “Zhaoxiang” (ZX, grown in Zhaoqing) cultivars according to the different growing regions in China (Wu et al., 2010; Wu et al., 2011). Under the long-term influence of internal plant factors and external conditions, the morphological traits and patchouli oil components and contents of different *P. cablin* cultivars differ (Li, Wu & Guo, 2011). According to the signature constituent of the leaf, *P. cablin* was classified into two different chemotypes: PA-type (ZNX and NX) and PO-type (PX and ZX) (Luo et al., 2003).

PA, a tricyclic sesquiterpene alcohol, is one of the main ingredients of patchouli oil. PA is used as an index ingredient in evaluating the quality of *P. cablin* medical materials and patchouli oil in different versions of the Chinese Pharmacopeia. PA shows anti-inflammatory (Xian et al., 2011), antiviral (Kiyohara et al., 2012), antiulcer (Liang et al., 2018), antiphotoaging (Feng et al., 2014) and antidepressant activities (Sah, Mathela & Chopra, 2011). With widespread attention and appreciation regarding the pharmacological activity and use of PA, the global demand for PA for medicinal, food, and daily cosmetic uses is increasing every year. Due to clinical and industrial needs, the demand for *P. cablin* exceeds the supply. The sources of PA include extraction from plants and chemical synthesis. Due to the complex three-dimensional structure of the five chiral carbon molecules of PA, its chemical synthesis is expensive and difficult with very low yields (Zhang et al., 2019c). *P. cablin* is the main biological source of PA, but the PA content of *P. cablin* is low. Hence, to increase the yield of PA, it is necessary to understand its biosynthetic mechanism in *P. cablin* with the specific goal of regulating the expression levels of the enzymes involved in PA synthesis.

**Figure 1:**
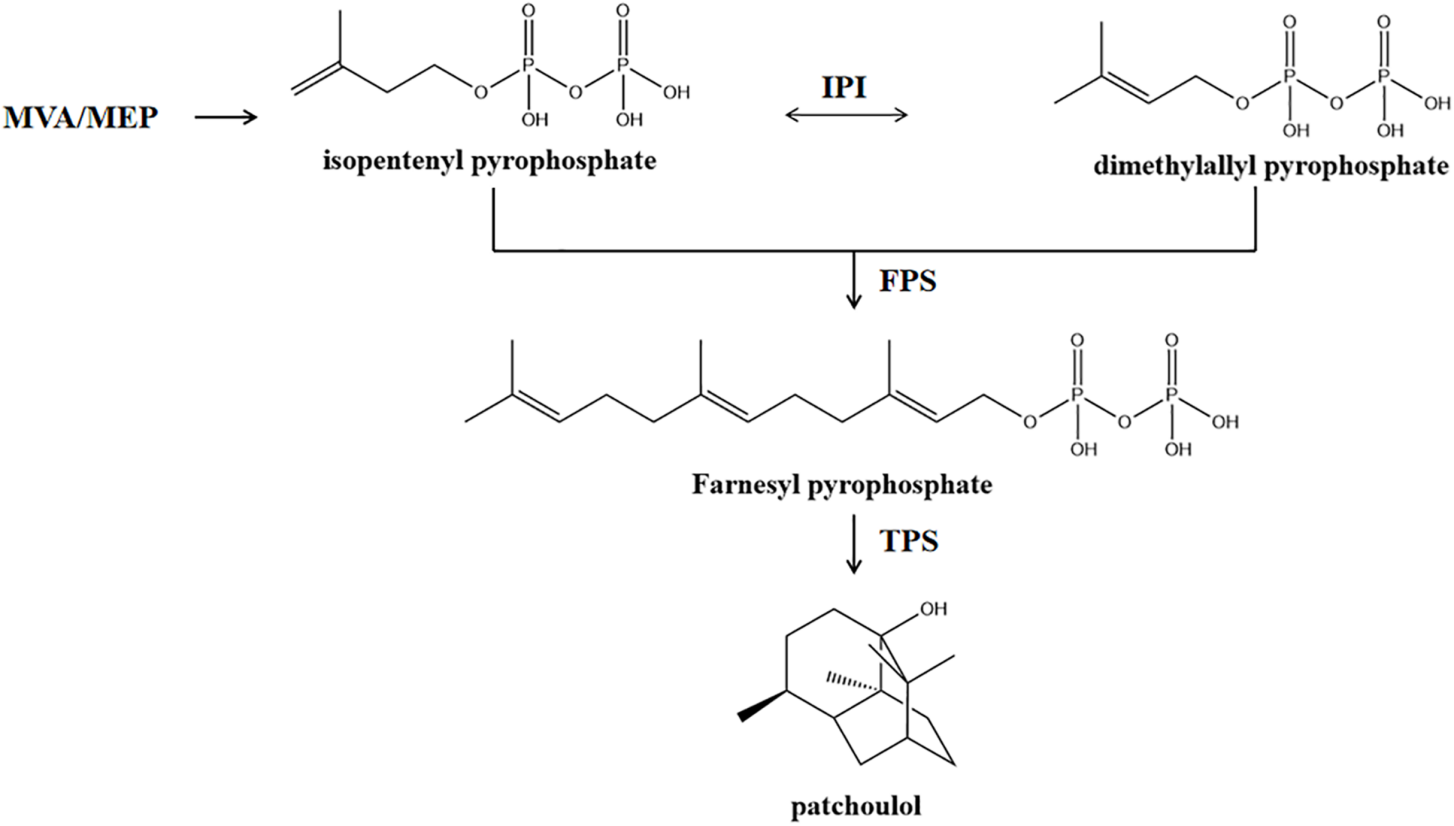
A simplified schematic representation of the plastidial MEP pathway and cytosolic MVA pathway to PA derived from IPP and DMAPP in *P. cablin*. IPP and DMAPP generated from the MVA or MEP pathway are catalyzed by FPS to form FPP with a C15 skeleton. FPP is cyclized by TPS to form PA carbon skeletons and then modified to form PA.

Terpenoids are a class of natural compounds with a five-carbon (C5) basic unit that are widespread in nature and mainly occur in plants as constituents of essential oils (Ludwiczuk, Skalicka-Woniak & Georgiev, 2017). On the basis of the C5 units, we can categorize terpenoids as hemiterpenes (C5), monoterpenes (C10), sesquiterpenes (C15), diterpenes (C20), sesterpenes (C25), triterpenes (C30), tetraterpenes (C35), and polyterpenes (>C40) (Ashour, Wink & Gershenzon, 2010). All of these compounds are found in plants, and PA along with all terpenoids are derived from C5-unit isopentenyl diphosphate (IPP), produced *via* the cytosolic mevalonate (MVA) and plastidic 2-C methyl-D-erythritol-4-phosphate (MEP) pathways (Chen et al., 2019). The MVA pathway uses acetyl-CoA as a raw material and condenses three molecules of acetyl-CoA to 3-hydroxy-3-methylglutaryl CoA (HMG-CoA) under the action of acetyl-CoA transferase (ACAT) and HMG-CoA synthase (HMGS). The transformation of HMG-CoA to MVA is catalyzed by HMG-CoA reductase (HMGCR). MVA forms IPP after two phosphorylation reactions mediated by mevalonate kinase (MVK) and phosphomevalonate kinase (PMK) and decarboxylation mediated by diphosphomevalonate decarboxylase (MVD). The IPP isomer dimethylpropene pyrophosphate (DMAPP) is generated through the action of isopentenyl pyrophosphate isomerase (IPI). DMAPP and its isomer IPP are the final products of the MVA pathway and are regarded as universal precursors of terpenoid biosynthesis. DMAPP/IPP biosynthesis *via* the MEP pathway in plastids is initiated by glyceraldehyde-3-phosphate and pyruvate. The condensation of glyceraldehyde-3-phosphate and pyruvate to produce 1-deoxy-D-xylulose-5-phosphate (DOXP) is catalyzed by 1-deoxy-D-xylulose-5-phosphate synthase (DXS). DOXP is reduced to MEP by 1-deoxy-D-xylulose-5-phosphate reductoisomerase (DXR), which is the rate-limiting step of the MEP pathway. Subsequently, the transformation of MEP to 2-C-methyl-D-erthritol 2,4-cyclodiphosphate (MEcDP) is catalyzed by MEP cytidyltransferase (ISPD), 4-diphosphocytidyl-2-C-methyl-D-erythritol kinase (ISPE), and 2-C-methyl-D-erythritol 2,4-cyclodiphosphate synthase (ISPF) in a three-step reaction. The transformation of MEcDP into 1-hydroxy-2-methyl-2-butenyl-4-diphosphate (HMBDP) is catalyzed by (E)-4-hydroxy-3-methylbut-2-enyl-diphosphate synthase (ISPG). Finally, the transformation of HMBDP to IPP is catalyzed by 4-hydroxy-3-methylbut-2-enyl diphosphate reductase (ISPH). IPP and DMAPP generated from the MVA or DXP pathway are catalyzed by farnesyl diphosphate synthase (FPS) to form farnesyl pyrophosphate (FPP) with a C15 skeleton (Deguerry et al., 2006), which is the common synthetic substrate of PA and other sesquiterpenoids (Fig. 1). FPP is cyclized by a different sesquiterpene synthase (TPS) to form different sesquiterpene carbon skeletons and then modified to form sesquiterpenes with various structures and functions.

Ribonucleic acid sequencing (RNA-Seq) is a modern technique for profiling the transcriptome by using deep-sequencing technologies that can reveal global transcriptional activity in any species at single-base resolution (Wang, Gerstein & Snyder, 2009). RNA sequencing is frequently used to evaluate the expression differences among different cultivars of the same species (Cao et al., 2018; Guo et al., 2018). In medicinal plants in particular, the application of RNA-Seq in different cultivars will help us to recognize the biosynthetic pathways of medicinal ingredients and to identify new genes related to abiotic stresses that may be exploited in molecular plant breeding (Shankar, Bhattacharjee & Jain, 2016; Chen et al., 2018; Zhao et al., 2019).

In view of the significant differences in the composition and content of patchouli oil extracted from different *P. cablin* chemotypes, it is of interest to search for potential differences in the PA biosynthetic pathway in different chemotypes. In the present study, PA and PO in the leaves of different chemotypes were analyzed by gas chromatography-mass spectrometry (GC-MS), and RNA-Seq technology was used to construct and sequence complementary DNA (cDNA) libraries from Chinese-cultivated *P. cablin* cv. PA-type NX and PO-type PX. The major purpose of this research was to identify differentially expressed genes (DEGs) associated with PA biosynthesis by comprehensive analysis of the transcriptome and GC-MS data of different *P. cablin* chemotypes with various bioinformatics tools. In addition, the significant DEGs related to PA biosynthesis identified in the RNA-Seq analysis were further validated by quantitative real-time polymerase chain reaction (qRT-PCR). The results increase our knowledge of the mechanisms underlying differences in PA biosynthesis among different *P. cablin* chemotypes.

## Materials & Methods

### Plant materials

Plants of Chinese-cultivated *P. cablin* cv. PO-type “Paixiang” and PA-type “Nanxiang” were grown in the *P. cablin* germplasm resource garden of Hainan University, located in Haikou, China (20°3′35″ N, 110°19′8″ E). Young, vigorous branches of two *P. cablin* chemotypes were selected for cutting propagation. Healthy rooted plantlets were transplanted to plastic pots after cutting for 30 days and cultivated in a greenhouse at 20–28 °C under natural sunlight. Fresh leaf tissues of 8-month-old *P. cablin* plants were collected from each cultivar. Individual leaves of each chemotype were sampled in triplicate and used for GC-MS and transcriptome analysis. For RNA extraction, all of the samples were immediately frozen in liquid nitrogen and stored at −80 °C. At the same time, fresh leaves of different chemotypes were obtained for essential oil isolation.

### Quantitative analysis of PA and PO in *P. cablin* leaves

Essential oil from *P. cablin* leaves was extracted by ultrasonic extraction (Yan et al., 2021). The extracts were dried over anhydrous sodium sulfate, and the resulting essential oils were stored at −10 °C prior to GC-MS analysis. Three individual replicates of each sample collected under the same conditions were analyzed.

GC-MS analysis was carried out on an Agilent 7890B-7000B GC-MS (Agilent Technologies, Santa Clara, CA, USA) and performed according to a previously described method (Yan et al., 2021). The PA content was quantified by an external standard curve, and the relative content of PO was also calculated based on the PA standard curve. The quantification of PA and PO is presented as the means of at least three replicates ± standard error.

### RNA isolation and Illumina sequencing

RNA extracted from the leaves of the two *P. cablin* chemotypes was isolated using the RNAprep Pure Plant Kit (DP441; TIANGEN, Beijing, China) following the manufacturer’s instructions. Total RNA integrity was checked by 1.2% agarose gel electrophoresis. The quality and concentration of extracted RNA were checked on an Agilent 2100 Bioanalyzer with an Agilent RNA 6000 Nano Kit (Agilent Technologies, Santa Clara, CA, USA). The purity of total RNA was detected by using a NanoDrop^TM^ ultraviolet spectrophotometer (Thermo Fisher Scientific, Waltham, MA, USA). Three biological replicates of all of the samples were performed to maximize the reliability of the statistical analysis.

Qualified total RNA was digested with deoxyribonuclease (DNase) and used for transcriptome sequencing. Transcriptome sequencing and the construction of the cDNA library were conducted by the OE Biotech Co., Ltd. (Shanghai, China). mRNA was enriched with oligo (dT) magnetic beads and then broken into short fragments by adding fragmentation buffer. The fragmented mRNA was used as a template to synthesize first-strand cDNA with a six-base random primer. The double-stranded reaction system was prepared to synthesize the second strand of cDNA, and a QiaQuick PCR Purification Kit (QIAGEN, Hilden, Germany) was employed to purify the double-stranded cDNA. End repair of the purified double-stranded cDNA was subsequently performed, followed by the addition of a poly(A) sequence and the ligation of adapters. Fragments of the desired size were selected by agarose gel electrophoresis. Finally, the acquired fragments were enriched by PCR amplification. After the constructed libraries were qualified on an Agilent 2100 Bioanalyzer (Agilent Technologies, Santa Clara, CA, USA), they were sequenced on the Illumina HiSeq X Ten platform (Illumina Inc., San Diego, CA, USA), and 150 bp paired-end reads were generated. The raw RNA-Seq data were deposited in the National Center of Biotechnology Information (NCBI) Sequence Read Archive (SRA, http://www.ncbi.nlm.nih.gov/Traces/sra/) under accession numbers PRJNA660501 and PRJNA660544.

### Transcriptome data filtration, assembly and gene function annotation

The original image data files acquired by high-throughput sequencing were transformed into raw reads by base calling and stored in FASTQ format, which contained the sequence information of the sequencing reads and the corresponding sequencing quality information. In view of the impact of the data error rate on the results, the raw reads were preprocessed using Trimmomatic software (Bolger, Lohse & Usadel, 2014) to eliminate adapters, low-quality reads (reads with more than 5% uncertain bases) and sequences with Phred quality scores below 20. After data processing, the raw sequences were converted into clean reads. The GC content and the Q30 (percentage of bases with a Phred value > 30) of the clean data were calculated. After the application of quality control, comparison and filtration procedures to the raw reads, Trinity software (Grabherr et al., 2011) was used to obtain transcript sequences *via* the paired-end splicing method. The longest transcript was chosen as the nonredundant unigene based on sequence length and similarity as a reference sequence for subsequent analysis. Diamond software (Benjamin, Chao & Daniel, 2015) was employed to annotate the functions of unigene sequences using the NCBI Nonredundant (NR) protein database, SwissProt protein database, euKaryotic Orthologous Groups of proteins (KOG) database, evolutionary genealogy of genes: Nonsupervised Orthologous Groups (eggNOG) database, and Kyoto Encyclopedia of Genes and Genomes (KEGG) database (Kanehisa & Goto, 2000), with an E-value of ≤ 10^−5^. HMMER software (Jaina et al., 2013) was used for alignment to the Pfam database to perform the functional analysis of unigenes. Gene Ontology (GO) functional classifications and annotations were examined by using WEGO software and Blast2GO, respectively (Ye et al., 2006).

### Analysis of the differentially expressed unigenes

Clean reads were aligned to the assembled transcripts using the Bowtie2 program (Langmead & Salzberg, 2012). eXpress software (Roberts, 2013) was used to calculate the expression abundance of unigenes *via* the fragments per kilobase per million mapped reads (FPKM) method (Trapnell et al., 2010). Differential expression analysis of two chemotypes of *P. cablin* samples was performed using read counts with the DESeq R package (Anders & Huber, 2012). A *P* value < 0.05 and fold change > 2 or fold change < 0.5 were set as the thresholds for significant differential expression. The overall distribution of differentially expressed unigenes in the two groups was visualized with an MA plot and a volcano plot. Genesis 1.8.1 was employed to perform hierarchical clustering and generate heatmaps. The GOseq R package was used to implement the GO enrichment analysis on the basis of the Wallenius noncentral hypergeometric distribution (Young et al., 2010). KEGG enrichment analysis was performed with the aid of KOBAS version 2.0.12 software (Mao et al., 2005).

### Validation of DEGs by qRT-PCR analysis

qRT-PCR was used to verify the repeatability and reliability of the RNA-Seq data. RNA purification and extraction were performed as described above. A RevertAid First Strand cDNA Synthesis Kit (Thermo Fisher Scientific, Waltham, MA, USA) was used to synthesize the cDNA used for qRT-PCR. For qRT-PCR validation, 12 unigenes involved in the terpenoid backbone biosynthetic and sesquiterpenoid biosynthetic pathways were randomly selected. Primer 5.0 software (Premier Biosoft International, Palo Alto, CA, USA) was used to design primer pairs for the amplification of selected genes. The total amount of cDNA in every reaction was normalized, and 18S ribosomal RNA (18S rRNA) was used as an internal reference for relative expression calibration. ChamQ Universal SYBR qPCR Master Mix (Vazyme Biotech, Nanjing, China) was used to conduct qRT-PCR with three replications on a Roche LightCycler®96 system (Roche, Switzerland). The 2^−∆∆CT^ method (Livak & Schmittgen, 2001) was used for the analysis of relative expression data. The primers used for qRT-PCR analysis are listed in Table S1. The selected gene sequences are listed in File S1.

### Simple sequence repeat and transcription factor identification

Microsatellite markers (SSRs, simple sequence repeats) were identified by using the MISA Tool (http://pgrc.ipk-gatersleben.de/misa). Due to the importance of transcription factors (TFs) in regulating gene expression, iTAK (Zheng et al., 2016) was used to align unigenes to its database to obtain TF annotation information.

## Results

### The contents of PA and PO from the Chinese-cultivated *P. cablin* cv. “Paixiang” and “Nanxiang”

To more precisely determine the differences in PA and PO contents among the Chinese-cultivated *P. cablin* cv. “Paixiang” and “Nanxiang,” a GC-MS approach was used. The data showed that *P. cablin* cv. “Paixiang” contained higher levels of PO (1.06 ± 0.03 mg.g^−1^, dry weight) than *P. cablin* cv. “Nanxiang” (0.59 ± 0.02 mg.g^−1^, dry weight). However, *P. cablin* cv. “Nanxiang” contained higher levels of PA (4.18 ± 0.06 mg.g^−1^, dry weight) than *P. cablin* cv. “Paixiang” (2.06 ± 0.07 mg.g^−1^, dry weight). To investigate the biosynthesis of major compounds, we proceeded with the analysis of the transcriptomes of the two *P. cablin* chemotypes.

### Transcriptome sequencing and *de novo* assembly

Six total RNA samples (PX-1, PX-2 and PX-3 for Paixiang and NX-1, NX-2 and NX-3 for Nanxiang) were isolated from different *P. cablin* chemotypes. RNA samples exhibiting concentrations of approximately 400–600 ng/µL, OD260/280 ratios ≥ 2.0, and RNA integrity numbers (RINs) of 9.0–10.0 were used for cDNA library construction. After the constructed libraries were qualified on an Agilent 2100 Bioanalyzer, the Illumina HiSeq X Ten platform was used to obtain a dataset of 6 cDNA libraries. The six libraries generated a total of 39.29 G raw bases. Approximately 36.83 G clean bases were obtained after filtering out low-quality sequences. NX produced 138,841,866 raw reads and 135,881,564 clean reads from three libraries, and PX produced 129,355,704 raw reads and 126,364,158 clean reads from three libraries. The Q30 values of all samples were distributed in the range of 92.73−93.91%, and the average GC content was 45.09%. The RNA-Seq data quality and quantity are presented in Table 1. The RNA-Seq results showed a low sequencing error rate, high single-base quality and no AT/CG separation (the GC and AT contents in each sequencing cycle were equal), indicating that the transcriptome sequencing results were of good quality. The sequencing results can be used for subsequent data assembly.

**Table 1:** Summary of Illumina transcriptome sequencing data for *Pogostemon cablin* included in this study.

Sample	Raw_reads	Raw_bases	Clean_reads	Clean_bases	Valid_bases (%)	Q30 (%)	GC (%)
NX1	41696522	6.11G	40710992	5.67G	92.75	93.25	44.81
NX2	42933824	6.29G	41888242	5.85G	92.94	93.40	44.82
NX3	54211520	7.94G	53282330	7.49G	94.34	93.91	44.95
PX1	44159282	6.47G	43142174	6.09G	94.08	92.85	45.47
PX2	44553200	6.53G	43540052	6.17G	94.47	92.96	45.48
PX3	40643222	5.95G	39681932	5.56G	93.32	92.73	45.01
Total	268197570	39.29G	262245722	36.83G			
Mean	44699595	6.55G	43707620	6.14G	93.65	93.18	45.09

**Note:**

Q30 represents the percentage of the number of bases with Phred scores > 30 in the raw bases to the total number of bases.

Trinity software was used to obtain transcript sequences *via* the paired-end splicing method. The longest transcript was selected as the nonredundant unigene based on the similarity and length of the sequence as a reference sequence for subsequent analysis. The longest transcript produced by *de novo* assembly contained 45,394 unigenes and had a length of 49,110,414 bp (as shown in Table 2). This transcriptome shotgun assembly project has been deposited at DDBJ/EMBL/GenBank under the accession GJHT00000000. The version described in this paper is the first version, GJHT01000000. The numbers of unigenes with lengths ≥ 500 bp and ≥ 1,000 bp were 30,841 and 17,731, respectively. The maximum and minimum lengths were 15,536 and 301 bp, respectively, with an average length of 1,081.87 bp. The N50 was determined to be 1,578 bp, which indicated that the quality of the sequence assembly was good. The length distribution and GC contents of the unigenes are shown in Fig. S1.

**Table 2:** Summary of the *de novo* assembly results for *Pogostemon cablin*.

Term	All (>300 bp)	>=500 bp	>=1,000 bp	N50	Total_Length	Max_Length	Min_Length	Average_Length
Unigene	45,394	30,841	17,731	1,578	49,110,414	15,536	301	1,081.87

### Gene annotation and functional classification

**Figure 2:**
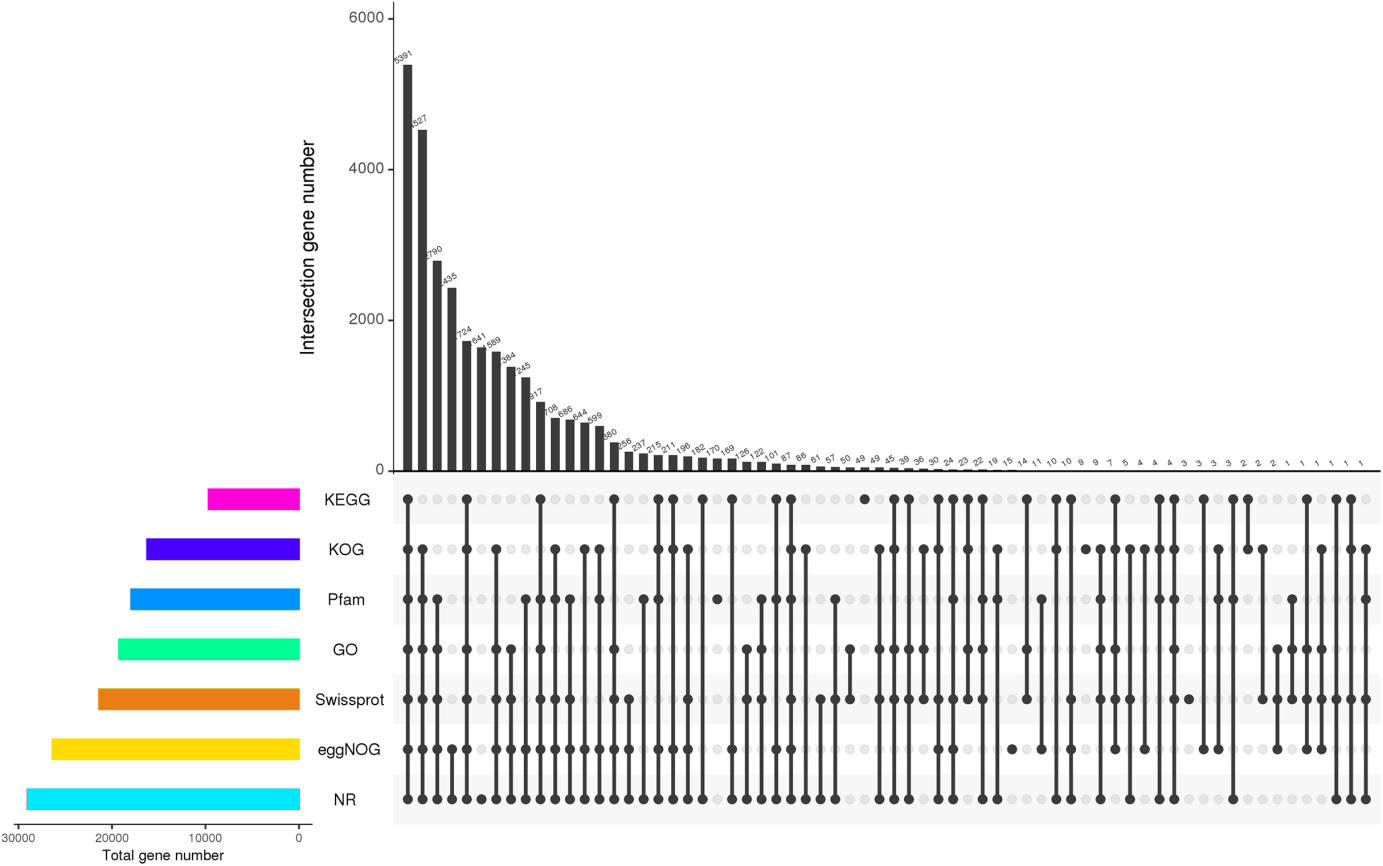
Annotation information obtained from seven different databases. The number on the upper bar graph represents the result of the intersection of the corresponding black-dotted databases in the matrix below, and the column on the left represents the number of genes annotated to each database.

**Figure 3:**
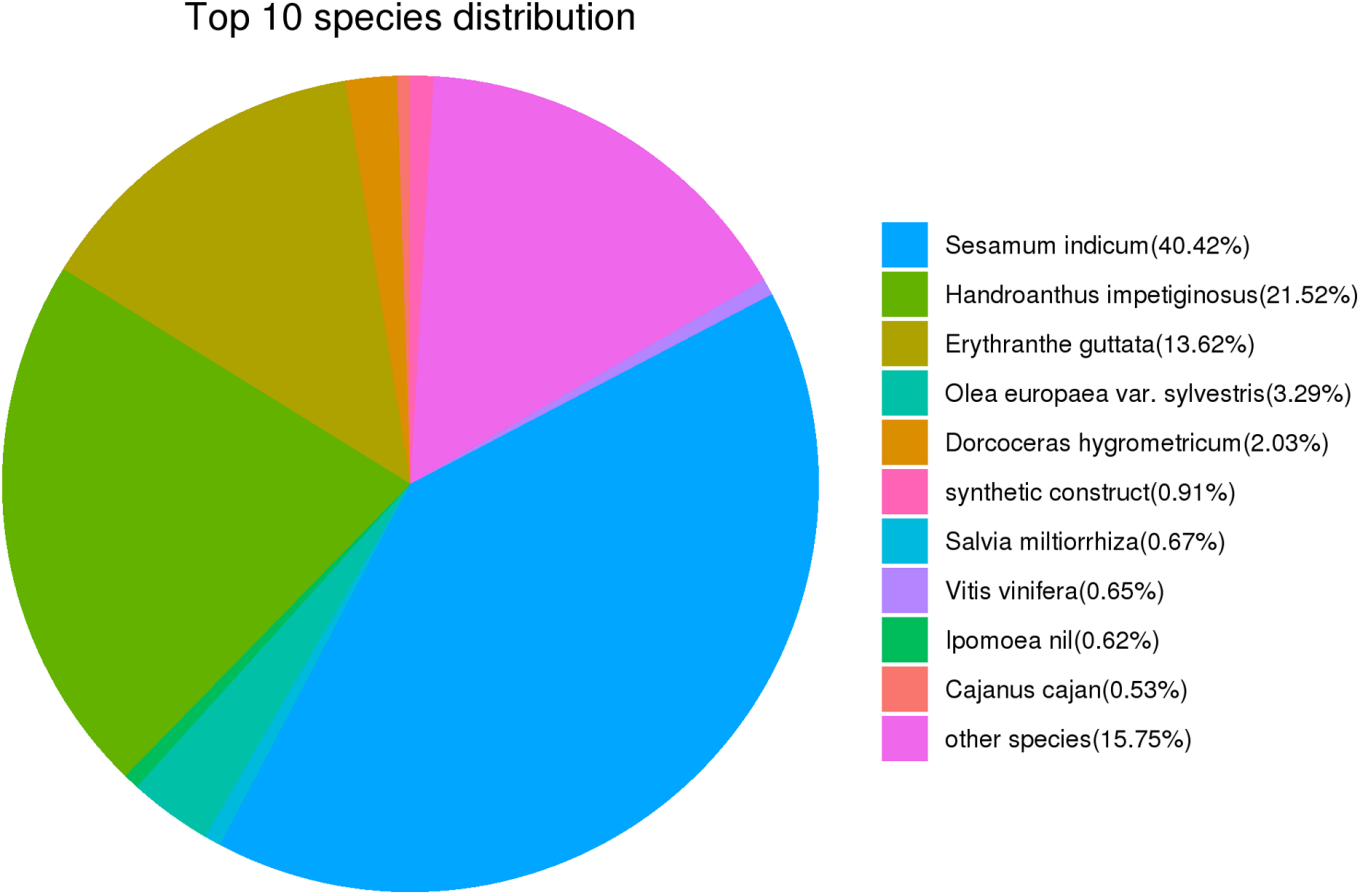
Characteristics of the sequence homology of *Pogostemon cablin* unigenes against the NR database-the top 10 species distribution.

For the functional identification of the assembled unigenes, all 45,394 unigenes were arranged against the KOG, NR, eggNOG, SwissProt, Pfam, KEGG and GO databases (as shown in Table 3). The results showed that 29,042 (63.98%), 21,381 (47.10%), 9,669 (21.30%), 16,241 (35.78%), 26,365 (58.08%), 19,253 (42.41%) and 17,953 (39.55%) unigenes were annotated to the NR, SwissProt, KEGG, KOG, eggNOG, GO, and Pfam databases, respectively. The UpSet plot of the annotations from each database is shown in Fig. 2. In *P. cablin* and other species included in the plant sequence homology study, the species unigene statistics were annotated with the help of the NR database (Fig. 3), and the five top-ranked species were *Sesamum indicum* (40.42%), *Handroanthus impetiginosus* (21.52%), *Erythranthe guttata* (13.62%), *Olea europaea* var. Sylvestris (3.29%) and *Dorcoceras hygrometricum* (2.03%). The reason for the relatively low homology observed with the Lamiaceae species transcripts and the high homology observed with *Sesamum indicum* may be related to the limited genetic information available for the Lamiaceae species in the public database. This outcome was also similar to the previous annotation in *P. cablin*.

**Table 3:** Unigene annotation statistics of the *Pogostemon cablin* transcriptome.

Annotation_Database	Annotated_Number	300 <= length < 1,000	length >= 1,000
NR	29,042 (63.98%)	12,968 (28.57%)	16,074 (35.41%)
Swissprot	21,381 (47.10%)	8,333 (18.36%)	13,048 (28.74%)
KEGG	96,69 (21.30%)	3,681 (8.11%)	5,988 (13.19%)
KOG	16,241 (35.78%)	6,225 (13.71%)	10,016 (22.06%)
eggNOG	26,365 (58.08%)	10,898 (24.01%)	1,5467 (34.07%)
GO	19,253 (42.41%)	7,367 (16.23%)	11,886 (26.18%)
Pfam	17,953 (39.55%)	5,467 (12.04%)	12,486 (27.51%)

**Figure 4:**
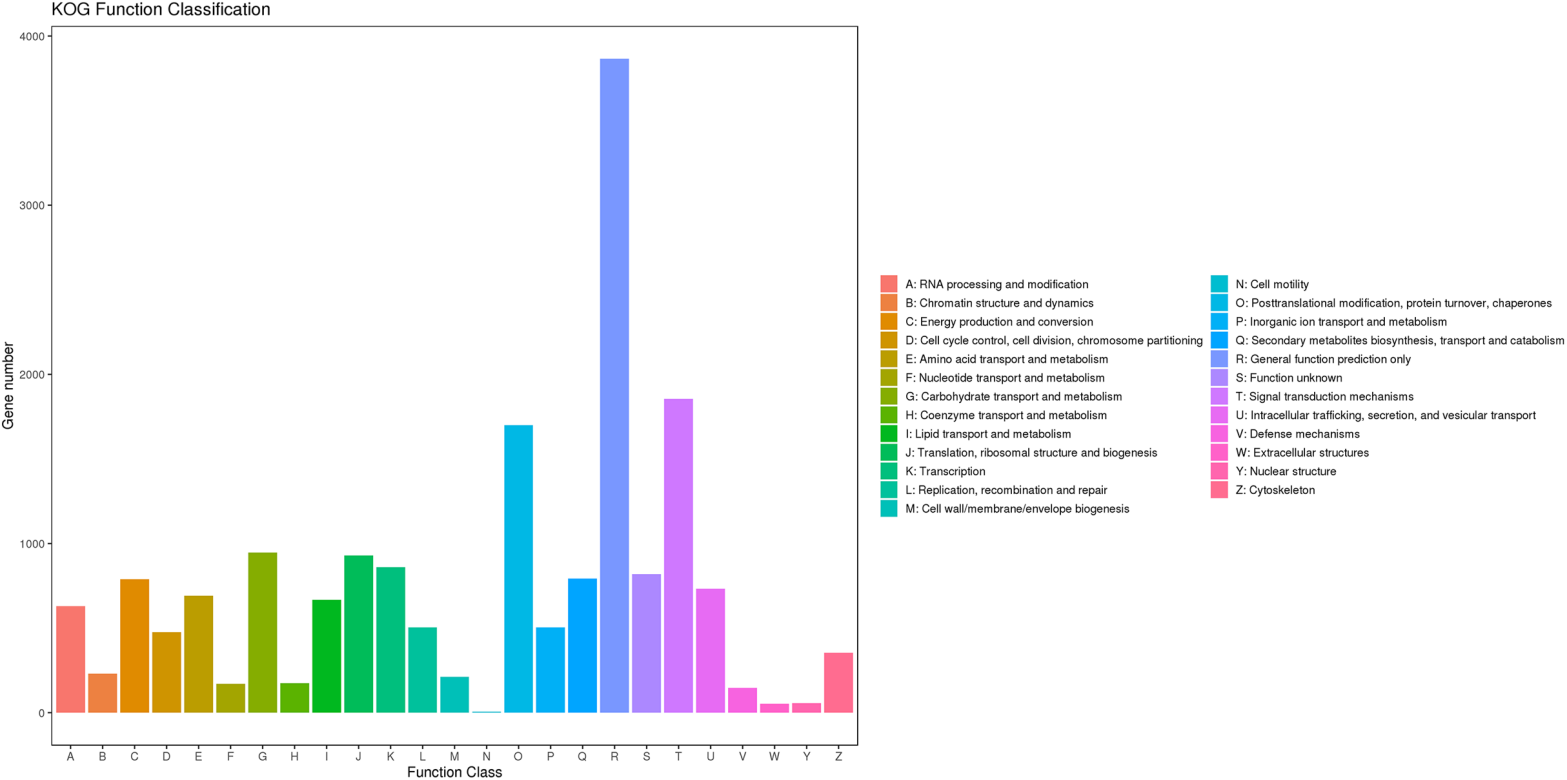
KOG functional classification of assembled unigenes.

When the assembled unigenes were aligned against the KOG database, a total of 16,241 matched unigenes were divided into 25 different functional categories (File S2; Fig. 4). The top three categories were “general function prediction only” (3,866, 23.80%), “signal transduction mechanisms” (1,855, 11.42%) and “posttranslational modification, protein turnover, chaperones” (1,700, 10.46%). Additionally, 792 (4.87%) unigenes were classified into the “secondary metabolite biosynthesis, transport and catabolism” category.

**Figure 5:**
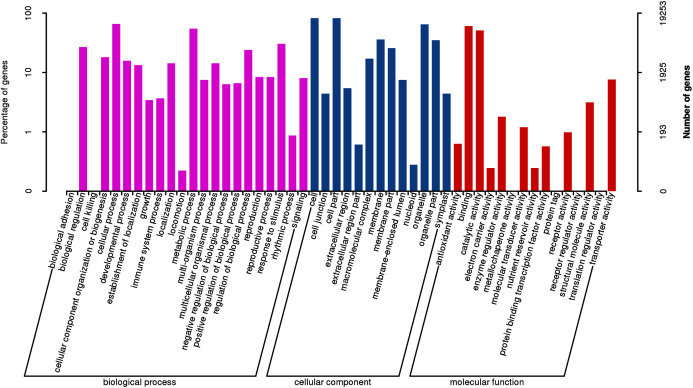
GO functional classification of assembled unigenes.

According to sequence homology, GO assignment analysis was performed (Fig. 5, File S3). A total of 19,253 unigenes were categorized according to 50 terms belonging to 3 major GO ontologies: molecular functions, biological processes and cellular components. In the biological process category, a total of 2,731 unigenes were categorized under 22 GO terms. A total of 6,735 and 9,788 unigenes were classified as belonging to the cellular component and molecular function categories under 10 and 11 GO terms, respectively.

**Figure 6:**
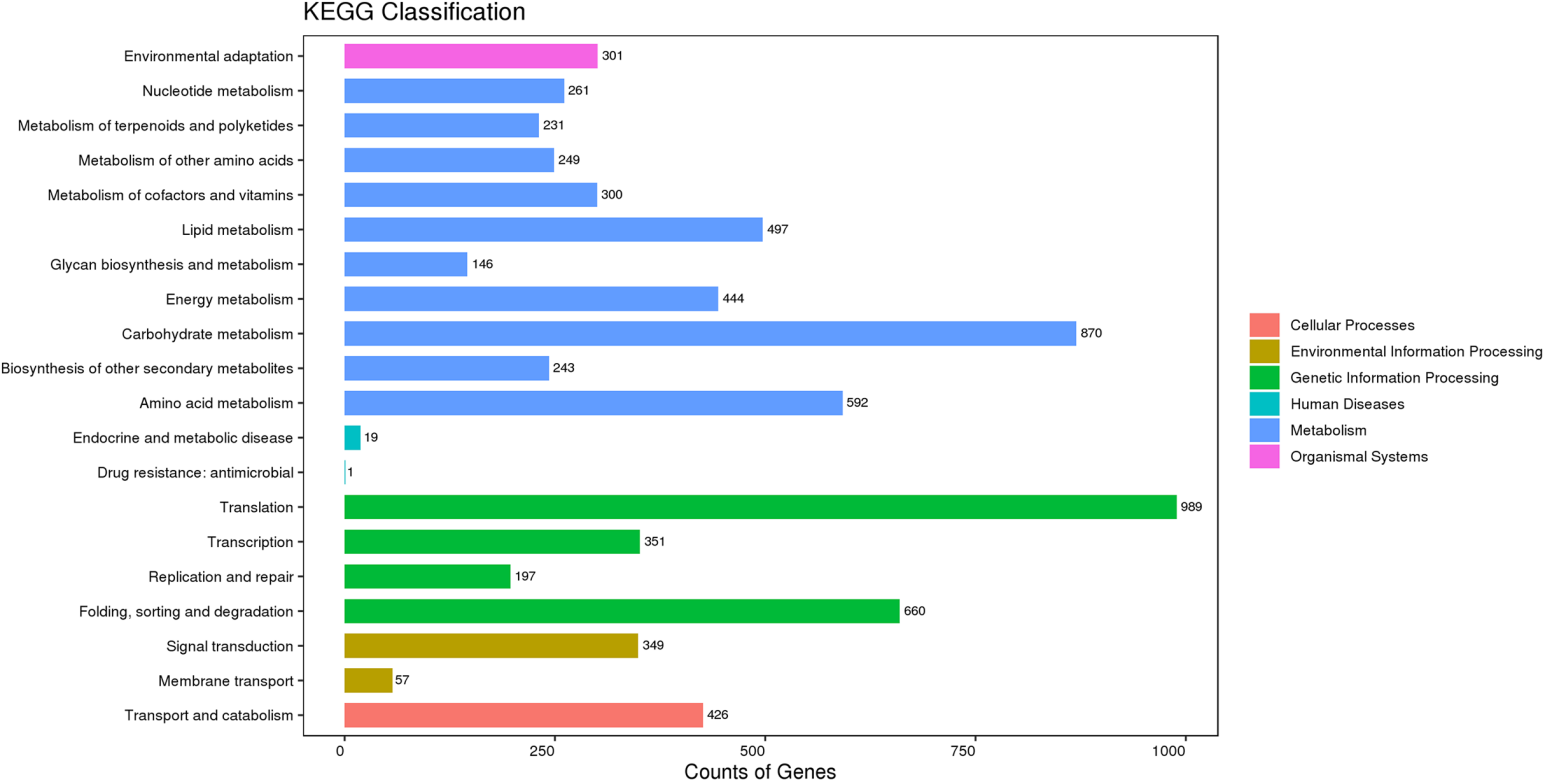
KEGG classification of the assembled unigenes at Level 2 (top 20). The horizontal axis represents the number of genes, the vertical axis represents the name of the Level 2 pathway, and the number on the right side of the column represents the number of genes annotated to the Level 2 pathway.

To identify the synthetic pathways of the bioactive components of *P. cablin*, the assembled unigenes were mapped to the KEGG annotation system. A total of 9,669 unigenes were mapped to 126 KEGG pathways (File S4). These KEGG pathways could be divided into six categories: metabolism, cellular processes, environmental information processing, genetic information processing, human diseases, and organismal systems (Fig. 6). The top five most enriched pathways were translation (989, 17.26%), carbohydrate metabolism (870, 15.18%), folding, sorting and degradation (660, 11.52%), amino acid metabolism (592, 10.33%) and lipid metabolism (497, 8.67%). Furthermore, 231 (4.03%) unigenes were involved in polyketide and terpenoid metabolism. Among these unigenes, 39 were mapped to diterpenoid biosynthesis (ko00904), 12 to monoterpenoid biosynthesis (ko00902), 23 to triterpenoid and sesquiterpenoid biosynthesis (ko00904) and 68 to terpenoid backbone biosynthesis (ko00900).

### Differential expression analysis of assembled unigenes

**Figure 7:**
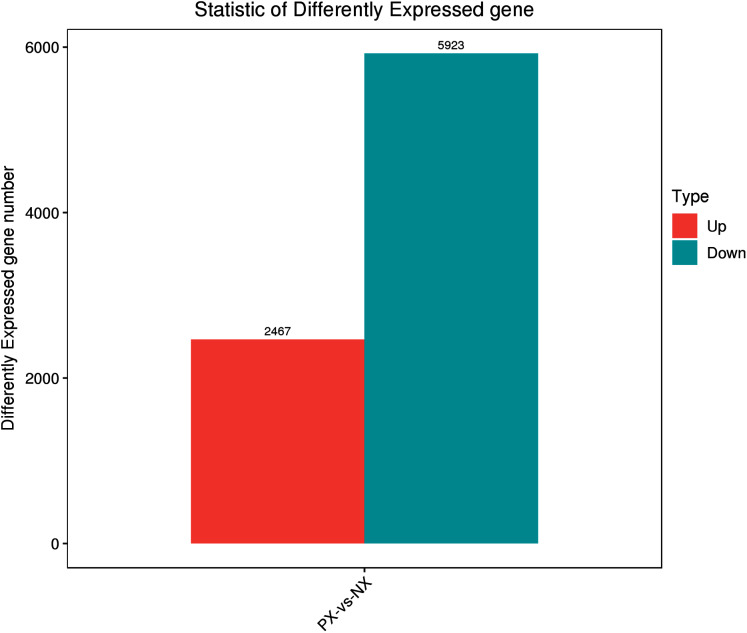
Statistics of differentially expressed genes between PX and NX.

**Figure 8:**
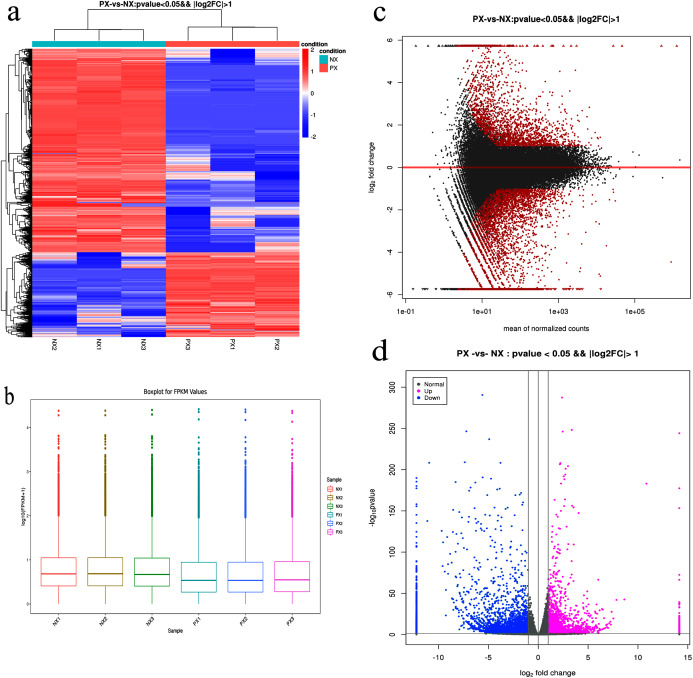
Expression analysis of the DEGs in PX *vs*. NX leaves. (A) Hierarchical clustering analysis. Different columns in the figure represent different samples, and different rows represent different genes. Colors from blue to red indicate gene expression from low to high, respectively. (B) Fragments per kilobase of transcript per million mapped reads (FPKM) boxplot. (C) MA plot. (D) Volcano plot. Each point in the MA plot and volcano plot represents a gene. The red points in the MA plot represent significant differentially expressed genes. In the volcano plot, the blue points represent downregulated genes, the purple points represent upregulated genes, and the gray points represent unchanged genes.

The clean reads from each library were aligned to unigenes using the Bowtie2 program. The alignment results (Table S2) showed that more than 88.61–91.25% of the reads from each library were matched to unigenes, while more than 84% were uniquely matched. To reveal the expression profile, the unigene expression level was calculated using eXpress software with the FPKM method. The DESeq R package was employed to characterize the DEGs in the chemotypes with |log_2_FoldChange| > 1 and q-value < 0.05, and a total of 8,390 DEGs were identified, including 5,923 downregulated and 2,467 upregulated genes in the two chemotypes (PX *vs*. NX) (File S5, Fig. 7). Among these DEGs, 49 were mapped to the metabolism of terpenoids and polyketides, among which 6 and 9 DEGs were mapped to sesquiterpenoid and triterpenoid biosynthesis (ko00909) and terpenoid backbone biosynthesis (ko00900), respectively. As shown in Fig. 8A, H-cluster (hierarchical cluster) analysis was performed for all the DEGs. The expression level per sample was evaluated according to the global discrete expression level (Fig. 8B). A MA plot (MA plots enable the visualization of variation in gene expression ratios as a function of the average signal intensity) was generated to display the total distribution of gene expression abundances and differential fold changes in PX *vs*. NX (Fig. 8C). A volcano plot was produced to show the differences in gene expression levels in PX *vs*. NX and their statistical significance (Fig. 8D).

Genes in the terpenoid backbone biosynthetic and sesquiterpenoid biosynthetic pathways with the largest expression differences between PX and NX were identified (Table 4). The largest difference in expression was detected in the TRINITY_DN22766_c0_g1_i8 gene, which encodes a protein similar to dammarenediol II synthase-like (GenBank: XP_011096562.1) identified in *S. indicum*; its expression level was 68.70-fold higher in NX in comparison with PX. However, its FPKM value was very low in both PX and NX. The second largest difference between the two chemotypes was found for the TRINITY_DN15930_c0_g1_i5 gene, which encodes patchoulol synthase variant 1 (GenBank: AHL24448.1) identified in *P. cablin*. Its average FPKM values in NX and PX were 641.92 and 15.81, respectively, and its expression level was 40.59-fold higher in NX in comparison with PX. The genes TRINITY_DN21681_c0_g1_i2, TRINITY_DN18757_c0_g1_i1 and TRINITY_DN28064_c0_g1_i8, which encode HMGS (identified in *S. indicum*), IPI (identified in *P. cablin*) and HMGCR (identified in *P. cablin*), were expressed at 0.58-, 0.60-, and 0.57-fold higher levels in PX in comparison with NX, respectively.

**Table 4:** Genes with the largest expression differences between PX and NX.

Gene	Expression in PX	Expression in NX	Diff	Annotation
TRINITY_DN22766_c0_g1_i8	0.15	10.21	68.70	Dammarenediol II synthase-like [*Sesamum indicum*]
TRINITY_DN15930_c0_g1_i5	15.81	641.92	40.59	Patchoulol synthase variant 1 [*Pogostemon cablin*]
TRINITY_DN14955_c0_g1_i1	28.04	116.72	4.16	Probable 1-deoxy-D-xylulose-5-phosphate synthase 2, chloroplastic [*Sesamum indicum*]
TRINITY_DN14252_c0_g1_i1	24.44	92.82	3.80	1-deoxy-D-xylulose 5-phosphate synthase, partial [synthetic construct]
TRINITY_DN30344_c0_g2_i7	14.60	50.03	3.43	1-deoxy-D-xylulose 5-phosphate synthase [*Plectranthus barbatus*]
TRINITY_DN22349_c0_g2_i1	5.40	18.00	3.33	Geranylgeranyl diphosphate reductase [*Actinidia chinensis* var. chinensis]
TRINITY_DN24538_c2_g3_i1	1.19	3.27	2.75	RecName: Full = Germacrene D synthase 2; AltName: Full = PatTpsBF2
TRINITY_DN20235_c0_g1_i3	3.79	10.37	2.74	Dammarenediol II synthase-like [*Sesamum indicum*]
TRINITY_DN28064_c0_g1_i8	40.55	24.24	0.60	HMG CoA reductase, partial [*Pogostemon cablin*]
TRINITY_DN21681_c0_g1_i2	84.34	49.32	0.58	Hydroxymethylglutaryl-CoA synthase [*Sesamum indicum*]
TRINITY_DN18757_c0_g1_i1	30.14	17.12	0.57	Isopentenyl diphosphate isomerase, partial [*Pogostemon cablin*]
TRINITY_DN30536_c2_g1_i3	6.08	3.28	0.54	Beta-amyrin synthase [*Sesamum indicum*]
TRINITY_DN27775_c0_g2_i2	1.55	0.54	0.35	Hypothetical protein MIMGU_mgv1a001796mg [*Erythranthe guttata*]

**Note:**

All annotations were based on BLASTx results at NCBI.

### Analysis of candidate genes involved in PA biosynthesis

PA, a tricyclic sesquiterpene alcohol, is one of the main ingredients in patchouli oil. PA biosynthesis consists of terpenoid backbone biosynthetic (Fig. S2) and sesquiterpenoid biosynthetic (Fig. S3) pathways. In this transcriptome analysis, 12 unigenes encoding six enzymes that participate in the MVA pathway were identified (File S6), including 2 ACAT (E2.3.1.9) genes, 1 HMGS (EC 2.3.3.10) gene, 4 HMGCR (EC 1.1.1.34) genes, 3 MVK (EC 2.7.1.36) genes, 1 PMK (EC 2.7.4.2) gene and 1 MVD (EC 4.1.1.33) gene. Additionally, we found 20 unigenes encoding seven enzymes that participate in the MEP pathway (File S6), including 8 DXS (EC 2.2.1.7) genes, 1 DXR (EC 1.1.1.267) gene, 1 ISPD (EC 2.7.7.60) gene, 3 ISPE (EC 2.7.1.148) genes, 2 ISPF (EC 4.6.1.12) genes, 1 ISPG (EC 1.17.7.1) gene and 4 ISPH (EC 1.17.7.4) genes. Additionally, 2 unigenes encoding IPI (EC 5.3.3.2), 1 unigene encoding FPS (EC 2.5.1.10) and 1 unigene encoding TPS1 were identified in this transcriptome. Based on the differential expression analysis of the PX vs NX transcriptomes, 5 unigenes (1 unigene encoding HMGS, 1 unigene encoding HMGCR, 1 unigene encoding IPI, and 2 unigenes encoding beta-amyrin synthase; Additional file 5) involved in the terpenoid backbone biosynthetic and sesquiterpenoid biosynthetic pathways were found to be upregulated in PX. In addition, 10 unigenes (1 unigene encoding (-)-germacrene D synthase (GERD), 2 unigenes encoding beta-amyrin synthase, 1 unigene encoding TPS1, 1 unigene encoding geranylgeranyl diphosphate synthase, GGPS, 1 unigene encoding geranylgeranyldiphosphate/geranylgeranyl-bacteriochlorophyllide a reductase, and 4 unigenes encoding DXS; Additional file 5) participating in the terpenoid backbone biosynthetic and sesquiterpenoid biosynthetic pathways were downregulated in PX.

### GO enrichment analysis of DEGs

**Figure 9:**
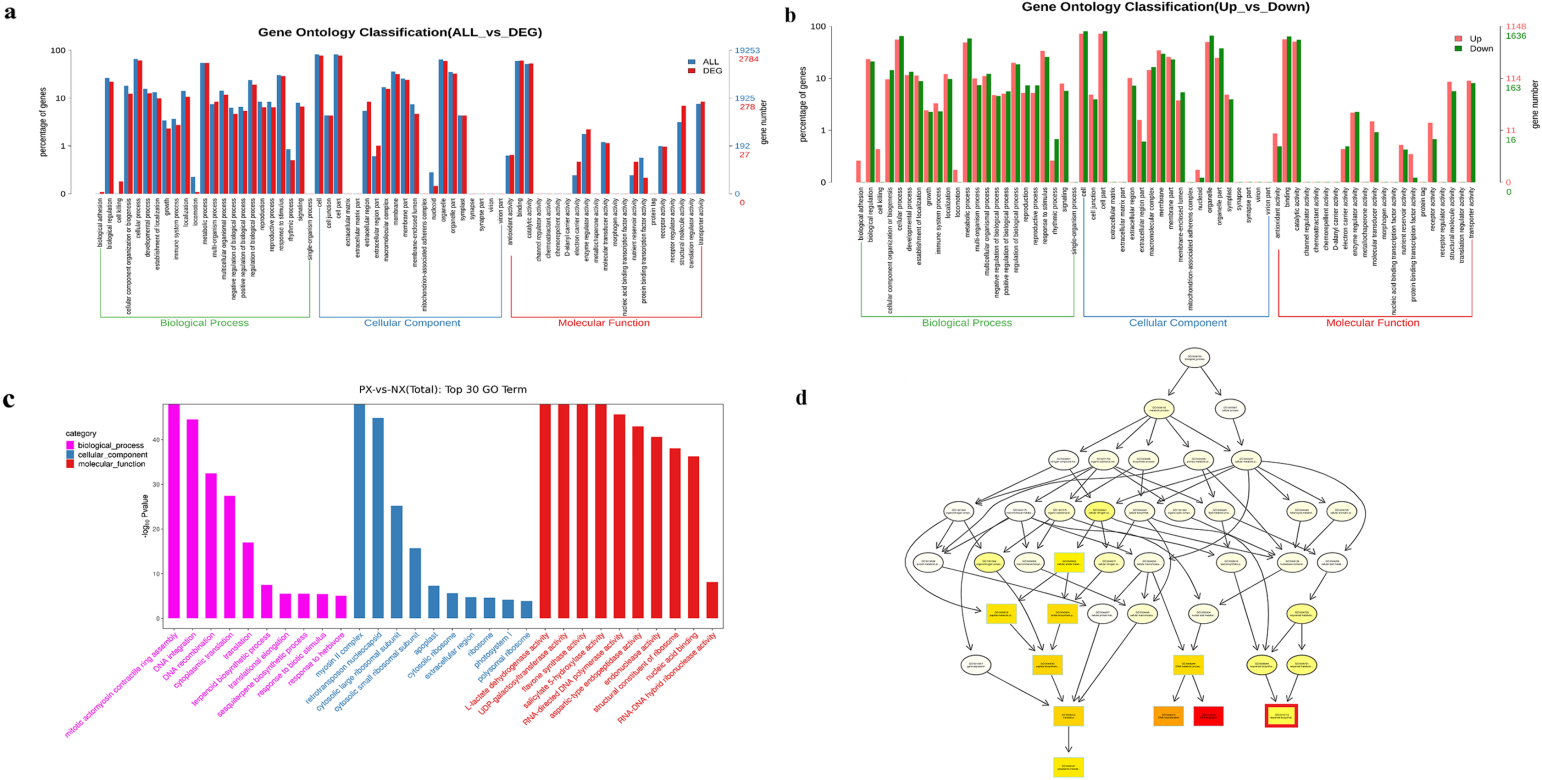
GO enrichment of DEGs. (A) ****GO enrichment of all DEGs. (B) GO enrichment of up-and downregulated DEGs. (C) ****Significantly enriched GO terms (*P* < 0.05). ****(D) Hierarchical tree graph of overrepresented GO terms in the biological process subcategory. The nodes are colored based on the q-value, and a darker color indicates a higher confidence level. The GO terms are presented at the horizontal node position.

To further study DEG functions, the GOseq R package was used to perform GO enrichment analysis of the DEGs. A total of 2,784 DEGs were annotated with GO terms and allotted to three main categories: biological processes, molecular functions, and cellular components (Fig. 9A). There were 1,148 upregulated DEGs and 1,636 downregulated DEGs identified between PX and NX (PX *vs*. NX, Fig. 9B). Figure 9C shows the 30 most enriched GO terms. The most upregulated genes were related to certain functional categories of these enriched terms, such as “mitotic actomyosin contractile ring assembly”, “myosin II complex”, “flavone synthase activity”, “salicylate 5−hydroxylase activity” and “structural constituent of ribosome”, whereas the downregulated genes were mainly associated with “DNA integration”, “DNA recombination”, “retrotransposon nucleocapsid”, “RNA-directed DNA polymerase activity”, “endonuclease activity”, “aspartic-type endopeptidase activity” and “nucleic acid binding”. The GO structure was described in the form of a directed acyclic graph (DAG) in which every GO term is drawn as a node and parentages are drawn as arrows. Terpene biosynthesis was significantly enriched (Fig. 9D).

### KEGG pathway enrichment analysis of DEGs

**Figure 10:**
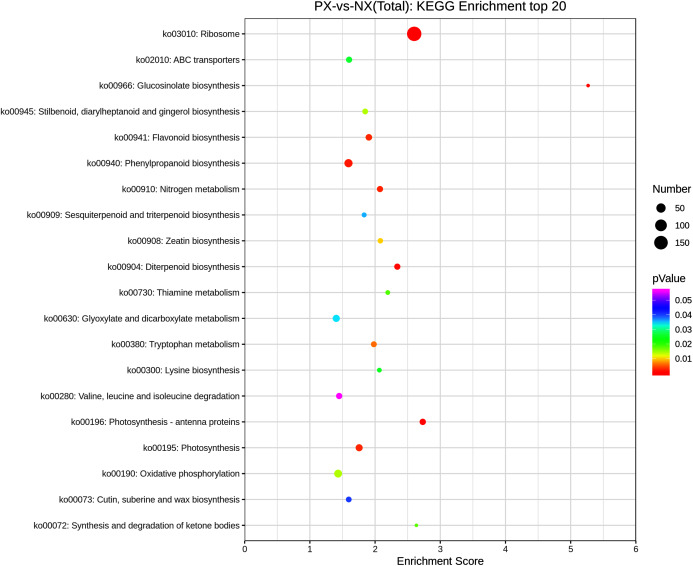
Scatter plot of enriched KEGG pathways of DEGs (top 20).

KEGG pathway enrichment analysis was conducted by using the 8390 DEGs to identify metabolic pathway differences in the two chemotypes. There were 816 DEGs mapped to 108 enriched KEGG pathways (File S7) in the transcriptomes from different *P. cablin* chemotypes, but only 19 pathways were significantly enriched (adjusted *P* < 0.05). Figure 10 presents the top 20 enriched pathways. Among the significantly enriched pathways, 13 DEGs were associated with diterpenoid biosynthesis (ko00904), 16 DEGs were assigned to flavonoid biosynthesis (ko00941), and 6 DEGs were associated with sesquiterpenoid and triterpenoid biosynthesis (ko00909). Among them, the *TPS1* gene was significantly enriched.

### DEGs related to TFs

**Figure 11:**
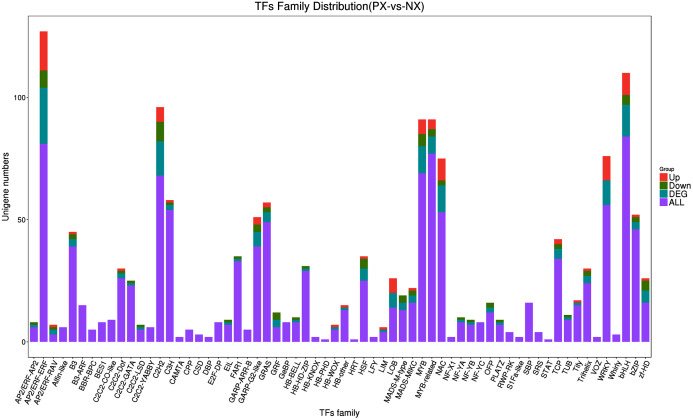
Identification of transcription factors in the two *P. cablin* chemotypes.

TFs are proteins that bind to specific DNA sequences and play a critical role in the regulation of gene expression by controlling the transcription of genetic information from DNA to RNA. iTAK was used to align the plant species unigenes to its own database to obtain TF annotation information. In total, 1,213 unigenes were annotated to the TF database, which were classified into 64 TF families (Fig. 11, File S8). Among these TFs, AP2/ERF (APETALA2/ethylene responsive factor), bHLH (basic helix-loop-helix), MYB (v-myb avian myeloblastosis viral oncogene homolog), MYB-related, WRKY, NAC (NAM, ATAF1/2, CUC1/2), GRAS, bZIP (basic region-leucine zipper), C2H2 and C3H genes represented the top 10 TF types in terms of abundance. A total of 23 unigenes encoding AP2/ERF proteins were differentially expressed in the leaves of the two chemotypes. Among these DEGs, 16 were upregulated and 7 were downregulated (PX *vs*. NX). Among the 13 differentially expressed bHLH genes, 4 were downregulated, while 9 were upregulated. There were 11, 7, 11, 4, 14, 2, and 3 DEGs encoding MYB, MYB-related, NAC, GRAS, C2H2, C3H, and bZIP family members, among which 5, 3, 2, 2, 8, 1, and 2, respectively, were downregulated and the other 6, 4, 9, 2, 6, 1, and 1 were upregulated. All 10 DEGs of the WRKY family were upregulated. In addition, 24 unigenes encoding Trihelix family TFs were identified in this study; one was upregulated, and two were downregulated. The sixteen unigenes encoding SBP family TFs showed no differential expression.

### Analysis of SSR information

**Figure 12:**
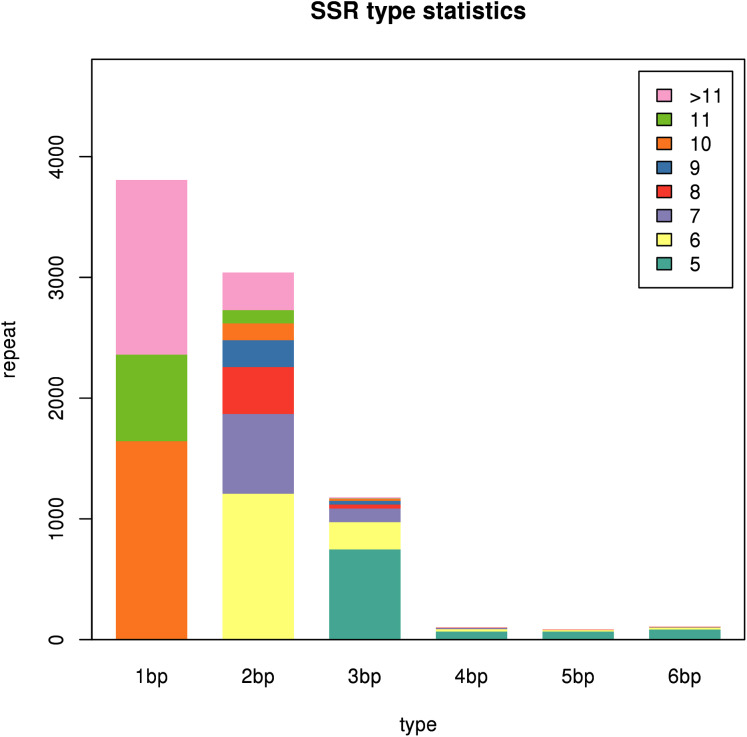
SSR types in the two *Pogostemon cablin* chemotype transcriptomes. The different colors depicted in the figure inset (upper right) represent the number of repeats of the repeating unit. The different colors depicted in the figure inset (upper right) represent the number of repeats of the repeating unit.

A total of 45,394 unigenes assembled in this study were used to explore potential microsatellites using MISA software. A total of 8,314 SSRs were identified in 6,825 unigenes, with a distribution frequency of 18.32%, among which 1,202 unigenes contained more than one SSR. The number of compound SSRs was 559 (Table 5). The distribution of SSRs is shown in Fig. 12. The largest fraction of the identified SSRs were mononucleotide repeats (3,806), followed by dinucleotide repeats (3,039) and trinucleotide repeats (1,178). Among the mononucleotide repeat motifs identified, the A/T repeat motif was the most abundant. AG/CT represented the most abundant dinucleotide motif, followed by AC/GT and AT/AT. The most abundant trinucleotide repeat motif was AAG/CTT, followed by CCG/CGG and ACC/GGT. The details of the SSR repeats are provided in File S9. Candidate SSR-specific primers were also designed and are provided in File S10.

**Table 5:** Summary of the SSR types in the transcriptomes of the two *Pogostemon cablin* cultivars.

Description	Number
Unigene number	45,394
Unigene size	49,110,414
SSRs number	8,314
Unigene number (SSR >= 1)	6,825
Unigene number (SSR >= 2)	1,202
Compound SSRs number	559
Distribution to different repeat type classes	Number of SSRs
Mono-nucleotide	3,806
Di-nucleotide	3,039
Tri-nucleotide	1,178
Tetra-nucleotide	101
Penta-nucleotide	83
Hexa-nucleotide	107

### qRT-PCR validation of DEGs from RNA-Seq analysis

**Figure 13:**
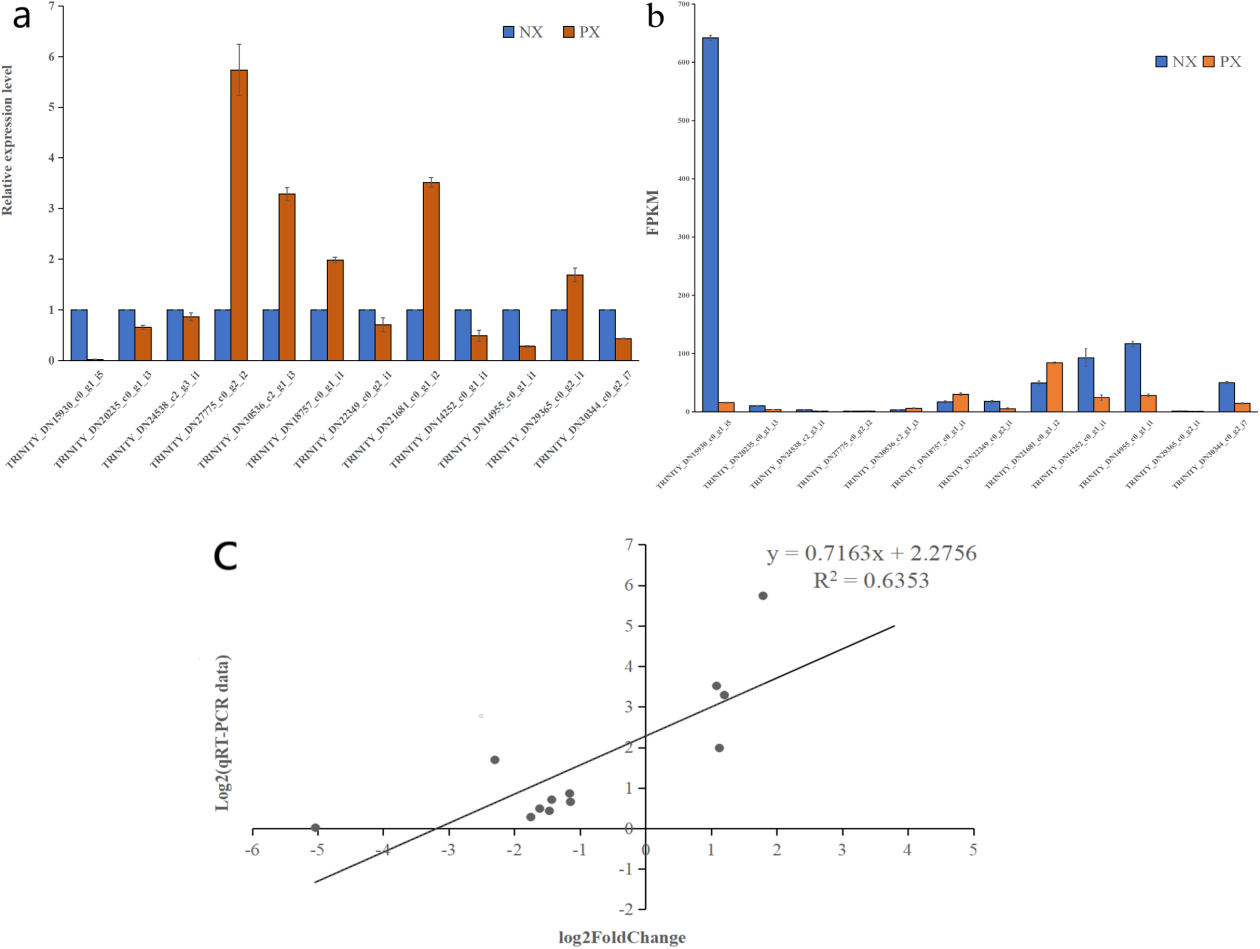
qRT-PCR verification of selected genes in the two *Pogostemon cablin* chemotypes. (A) qRT-PCR was performed to determine relative expression levels. (B) ****Expression patterns of 12 unigenes based on FPKM values. The error bars represent the standard deviations from three replicates. (C) Correlation plot of the RNA-Seq results (RPKM) and qRT-PCR (2^−ΔΔCt^) results. The R^2^ value represents the correlation between the RNA-Seq and qRT-PCR results.

To verify the DEG expression pattern obtained *via* RNA-Seq analysis, qRT-PCR was used to evaluate the expression levels of 12 unigenes involved in the terpenoid backbone biosynthetic and sesquiterpenoid biosynthetic pathways in PX and NX (Fig. 13). The expression levels (File S11) of eleven unigenes among the selected genes were roughly in accordance with those inferred from the FPKM data from RNA-Seq (Figs. 13A, 13B). The correlation between the qRT-PCR and RNA-Seq measurements was evaluated, and the R^2^ value was 0.6353 (Fig. 13C). The results of this research confirmed the reliability of the transcriptome profile data estimated from the RNA-Seq data.

## Discussion

As an important raw material for more than 30 types of patented Chinese medicines, *P. cablin* has a significant effect on the treatment of gastrointestinal cold, heat stroke, vomiting and diarrhea (Yan et al., 2021). Furthermore, patchouli oil contains abundant PA and PO and is an important ingredient used as a base to provide long-lasting properties to scents in the perfume industry (Zhang et al., 2018). Nevertheless, although PA from *P. cablin* has extremely high medicinal value, the functional genes associated with PA biosynthesis have not yet been fully elucidated. RNA-Seq has been frequently used to evaluate the expression differences among different cultivars of the same species. For instance, Zhou et al. (2019) analyzed the transcriptome and metabolome of *Pennisetum purpureum* cultivars with different colored leaves to study the molecular mechanisms associated with the biosynthesis and regulation of anthocyanins that regulate pigmentation in this species. Wang et al. (2017) analyzed the transcriptome and proteome of fruit pulp from the blood orange “Zaohong” and the navel orange “twenty-first century” to study *Citrus sinensis* quality-related molecular changes during consecutive developmental periods. In medicinal plants in particular, the application of RNA-Seq in different cultivars will help us to identify the biosynthetic pathways of medicinal ingredients and new genes related to abiotic stresses that may be exploited in molecular plant breeding.

### Transcriptome sequencing of two *P. cablin* chemotypes

With the development and maturity of next-generation and third-generation sequencing technologies, RNA-seq has been used to study the expression levels of key enzyme genes of a large number of medicinal plants in the biosynthesis of bioactive components, revealing the biosynthetic pathway and its regulatory mechanism of secondary metabolites (Wang et al., 2021; Sun et al., 2021). There are 14 projects in the NCBI SRA relating to *P. cablin*, containing more than 100 runs in total. He et al. (2016a) investigated the stem and leaf transcriptome characteristics of *P. cablin* cultivated in Yangchun using the Illumina HiSeq 2000 platform and identified 17 putative homologs of PA and PO biosynthesis-related genes. Chen et al. (2019) used PacBio Iso-Seq technology to generate the full-length transcriptome of *P. cablin* cultivated in Zhanjiang from leaf and stem tissues, in which 102 transcripts were annotated as encoding 16 enzymes related to PA biosynthesis. In addition, the draft genome and complete chloroplast genome sequences of *P. cablin* have also been reported, which would promote phylogenetic, population and genetic engineering research investigations of *P. cablin* (He et al., 2016b; 2016c). The *P. cablin* genome has also recently been updated (He et al., 2018). However, throughout the existing transcriptome studies on *P. cablin*, most of the studies were biased towards the Chinese-cultivated *P. cablin* cv. “Paixiang”, “Zhaoxiang” and “Zhanxiang”, and there have been few reports on Chinese-cultivated *P. cablin* cv. “Nanxiang”. *P. cablin* is widespread in southern China and is classified into four cultivars (Wu et al., 2010). Different cultivars of the same species are significantly different in terms of their quality and bioactive components when cultivated at different locations (Swamy et al., 2015). Chinese-cultivated *P. cablin* was divided into PA-type and PO-type based on differences in their volatile oil composition and content. Due to urban area expansion, arable land compression and variety degradation, the planting area of *P. cablin* cv. “Paixiang” and “Zhaoxiang” has drastically decreased. *P. cablin* cv. “Nanxiang” has become the main source of *P. cablin* herbs in China. *P. cablin* cv. “Nanxiang” is a typical PA-type *P. cablin*, and its PA content is much higher than that of *P. cablin* cv. “Paixiang” and “Zhaoxiang”. GC-MS analysis of PO-type Paixiang and PA-type Nanxiang in this study showed that the PA content was significantly higher in NX than in PX, while the PO content showed the opposite phenotype. This result was consistent with the results of previous research (Liu et al., 2002; Luo et al., 2003; Huang et al., 2016) and provides good experimental materials and novel ideas for the in-depth study of the PA biosynthetic pathway and mechanism of molecular regulation. Although the genome sequence of *P. cablin* has been updated at present, structural annotation and functional annotation information have not been disclosed. This severely hampers an understanding of the biosynthetic mechanisms of its active compounds. In particular, patchouli oil is in short supply worldwide, and the production of PA is even less. Only by understanding the biosynthetic pathways and regulatory mechanisms of PA can we improve the production of patchouli oil and PA to meet consumer demand by means of metabolic engineering. Recently, a number of studies have focused on individual key enzyme genes and transcription factors to elucidate the biosynthetic mechanism of PA (Yan et al., 2021; Zhang et al., 2019b, 2019c; Yu et al., 2015; Wang et al., 2019; Chen et al., 2020; Li et al., 2021). There are also some reports on the regulation of the synthesis of PA by exogenous plant hormones, circadian rhythms and developmental stages (Wang et al., 2019; Chen et al., 2019; Ouyang et al., 2016; Liu et al., 2018). However, understanding the biosynthetic pathway and molecular regulation mechanism of PA is the key to effectively increasing its production. Transcriptome analysis of medicinal plant cultivars with significant differences in the accumulation of bioactive substances can better elucidate their biosynthetic pathways and potential molecular regulation mechanisms. Here, we selected cultivars with low and high PA accumulation for transcriptome analysis to provide useful information for further studies on *P. cablin* and contribute to the elucidation of transcriptional regulation of the PA biosynthetic pathway. The reference genome for transcriptome sequencing needs not only sequence information but also corresponding structural annotation and functional annotation information. Currently, only genome sequence information (PRJNA471952) of *P. cablin* has been published, and no gene functional annotation information has been published. We also assembled the transcriptome based on reads and genome alignment information to obtain new transcripts for analysis. However, due to cultivar differences, the efficiency of genome alignment is not high. To fully discover genes related to PA biosynthesis, we chose the *de novo* assembly method for this transcriptome analysis.

In our transcriptome analysis, the two chemotypes generated 36.83 G clean bases, and 45,394 unigenes were annotated to seven databases. A total of 8,390 DEGs were identified between the cultivars, including 2,467 upregulated and 5,923 downregulated unigenes in PX. The functional annotation and KEGG pathway assignment revealed 231 unigenes allocated to the “metabolism of terpenoids and polyketides”; among these unigenes, 68 were allocated to terpenoid backbone (ko00900), 23 to sesquiterpenoid and triterpenoid (ko00909), 12 to monoterpenoid (ko00902) and 39 to diterpenoid (ko00904) biosynthesis. Comparative analyses of the RNA-Seq data from PX and NX leaves showed that 8,390 DEGs were differentially expressed, including 2,467 upregulated and 5,923 downregulated genes between NX and PX (PX *vs*. NX). Among these DEGs, 49 were mapped to the metabolism of terpenoids and polyketides, including 13 DEGs involved in diterpenoid (8 upregulated, 5 downregulated), 2 DEGs involved in monoterpenoid (1 upregulated, 1 downregulated), 6 DEGs involved in sesquiterpenoid and triterpenoid (2 upregulated, 4 downregulated) and 9 DEGs involved in terpenoid backbone (3 upregulated, 6 downregulated) biosynthesis.

### Identification of genes involved in PA biosynthesis

All terpenoids, including PA, originate from DMAPP and its isomer IPP *via* the MEP and MVA pathways. In the two evaluated *P. cablin* chemotype transcriptomes, 36 candidate unigenes involved in PA biosynthesis were identified, including 12 unigenes encoding six enzymes in the MVA pathway, 20 unigenes encoding seven enzymes in the MEP pathway, 2 unigenes encoding IPI, 1 unigene encoding FPS and 1 unigene encoding TPS1. The *TPS1* gene, in particular, showed significant differential expression in PX and NX, as it was expressed 40.59-fold higher in NX than in PX. According to BLASTx results at NCBI, this gene encodes patchoulol synthase variant 1 identified in *P. cablin*. Patchoulol synthase, a sesquiterpene synthase, is a key enzyme for the synthesis of PA in *P. cablin*. It can catalyze the sesquiterpene precursor FPP to form patchoulol and other sesquiterpenoids (Frister et al., 2015). This result was consistent with the differential accumulation of PA among the two *P. cablin* chemotypes. The GC-MS results of PX and NX leaves showed that the PA in NX was significantly higher than that in PX, which may be related to the overexpression of TPS1 in NX compared to PX. In addition, qRT-PCR analysis of the DEGs in the terpenoid backbone and sesquiterpenoid biosynthetic pathways showed that the DEG expression tendency was consistent with the transcriptome sequencing results. This research offers novel insights into the biosynthetic regulation of bioactive compounds in different *P. cablin* cultivars on a molecular basis and provides valuable resources for the potential metabolic engineering of this important medicinal plant.

Recently, genes involved in PA biosynthesis, such as *PcHMGCR* (Zhang et al., 2019c), *PcIPI* (Yan et al., 2021), and *PcFPS* (Zhang et al., 2019b), have been successfully isolated from *P. cablin* based on transcriptome data by our team, enabling us to elucidate the biosynthesis of PA and to produce increased PA yields in *P. cablin* through genetic engineering. Some genes (*DXS*, *GGPPS*, *CPPS*, etc.) of the terpene biosynthetic pathway have recently been successfully overexpressed in *Salvia* spp. to increase terpenoid yield (Vaccaro et al., 2020; Shi et al., 2016; Kai et al., 2011). This is a feasible strategy for enhancing the production of natural products by manipulating a number of biosynthetic genes at regulatory points. Wu et al. (2006) added both the *TPS* gene and the avian *FPS* gene to the chloroplast transit peptide sequence to achieve FPPS and TPS protein redirection in tobacco. It was also shown that the content of PA in the transformed plants reached 0.5 μg/g (fresh weight). Compared with plants, microorganisms exhibit several advantages in producing terpenoids, such as land savings, fast growth, and controllable culture conditions (Vickers et al., 2017). With the development of metabolic engineering and synthetic biology, many valuable plant-derived terpenoids have been produced in microbial cell factories (Ma et al., 2019). This method mainly increases the yield of PA by repressing the limiting steps and competitive steps, reducing industrial production costs and increasing yields. Recently, based on the MVA pathway or MEP pathway, heterologous production of PA has been accomplished by introducing the PA synthase gene in *Saccharomyces cerevisiae* (Ma et al., 2019) and *Corynebacterium glutamicum* (Henke et al., 2018) as well as in the moss *Physcomitrella* patens (Zhan et al., 2014) and eukaryotic microalga *Chlamydomonas reinhardtii* (Lauersen et al., 2016). In short, on the basis of a clear understanding of the biosynthetic pathway of PA, we can enhance the flux of the MVA/MEP pathway by overexpressing the key enzyme genes in this pathway and enhance the terpene synthetic enzymes or other rate-limiting enzymes related to terpene synthesis through overexpression or protein engineering to increase the production of PA.

### Differentially expressed TFs might be involved in regulating the synthesis of PA in *P. cablin*

TFs are proteins that specifically bind to the enhancer or promoter region of target genes to control their transcription, thereby playing a regulatory role in plant growth and development, secondary metabolite biosynthesis and responses to the external environment (Winkel-Shirley, 2001). The transcriptional activation of synthetic genes by TFs is one of the most important regulatory links in plant secondary metabolism. By activating the expression of multiple synthetic genes in plant secondary metabolite synthetic pathways, TFs can effectively initiate or shut down the secondary metabolic synthetic pathway, thereby regulating the synthesis of specific secondary metabolites. In recent years, researchers have also tried to use metabolic engineering strategies to increase the production of plant bioactive secondary metabolites through single key-limiting genes or transcription factors, providing strong evidence that it is feasible to boost secondary metabolism in crop and medicinal plants (Lu, Tang & Li, 2016; Farré et al., 2014). Thus far, studies have shown that there are 6 main TF families involved in terpene synthesis, including the AP2/ERF, bHLH, MYB, NAC, WRKY and bZIP families (Xu et al., 2019). For instance, the *AaERF1, AaERF2* and *AaTAR1* genes of the AP2/ERF family are involved in regulating the synthesis of artemisinin in *Artemisia annua* (Yu et al., 2012; Tan et al., 2015). The bHLH family is one of the largest TF families in plants and plays an important role in plant growth, development and secondary metabolism (Zhu et al., 2020). Many studies have found that MYC genes (Hong et al., 2012; Shen et al., 2016) of the bHLH family are involved in the regulation of the jasmonic acid signaling pathway, which has been proven to be involved in the regulation of terpene biosynthetic genes in plants. AtWRKY18, AtWRKY40, and AtMYC2 TFs were heterologously expressed in *Salvia sclarea* hairy roots and proved to regulate the expression of several genes encoding enzymes of the MEP-dependent pathways in a coordinated manner, especially DXS, DXR, GGPPS and CPPS (Alfieri et al., 2018). Similarly, the plant MYB TF family not only participates in the physiological process of plant stress resistance but also responds to the JA signaling pathway, and its members are important TFs regulating the synthesis of flavonoids and terpenoids (De Geyter et al., 2012; Zhou et al., 2017). The *MYB36/MYB9b* and *MYB1* genes are involved in the regulation of tanshinone in *Salvia miltiorrhiza* (Ding et al., 2017; Zhang et al., 2017) and artemisinin in *Artemisia annua* (Matías-Hernández et al., 2017), respectively, in which they play a promoting role. In *P. cablin*, four TF families have been reported, including PatMYB46 in the MYB family, PatMYC2b1 and PatMYC2b2 in the bHLH family, PatGT-1 in the TH family and SPL10 in the SBP family. Yu et al. (2015) found that overexpression of SPL10 in *P. cablin* could increase the synthesis of PA. Wang et al. (2019) found that PatMYC2b1 and PatMYC2b2 could bind to the PatJAZ6 protein to regulate PA synthesis. Chen et al. (2020) found that overexpression of PatMYB46 in *P. cablin* could increase the content of PA. Li et al. (2021) found that overexpression of PatGT-1 in *P. cablin* could reduce the content of PA and revealed that PatGT-1 negatively regulates PA biosynthesis. Recently, the molecular dynamics of *P. cablin* have been gradually revealed, especially the genes controlling the synthesis of PA (Tang et al., 2019). However, there is limited information on the regulation of its TFs to date, especially PA biosynthesis in *P. cablin*.

In total, 1,213 unigenes were annotated to the TF database in this research and were classified into 64 TF families. Among these TFs, 23 AP2/ERF-ERF, 3 Trihelix, 13 bHLH, and MYB (11) and MYB-related (7) genes were differentially expressed between the PX and NX transcriptomes. The subsequent expression analysis revealed that these genes were significantly upregulated and downregulated in the leaves of different *P. cablin* chemotypes and might play critical roles in PA biosynthesis. Furthermore, 11, 3 and 10 DEGs encoding NAC, bZIP, and WRKY family members, respectively, were identified, among which 2, 2 and 0 were downregulated and 9, 1 and 10 were upregulated. In addition, 16 unigenes encoding SBP family members were found in this study, but no differential expression was observed. At present, related studies have mainly focused on the cloning, expression and basic functions of plant TFs. Since there is no mature genetic transformation system in *P. cablin*, it is difficult to identify the functions of specific PA-related genes in the plant itself. Therefore, identifying a single key TF is more efficient than studying a large number of structural genes and can provide new ideas and methods for improving PA production. With the continuous development and improvement of transgenic technology and protein interaction methods, it is easier to obtain TFs and their complexes that are useful for *P. cablin*. In this way, we can further understand the synthetic pathway and molecular regulation mechanism of PA.

### SSR distribution and frequency in the *P. cablin* transcriptome

At present, Chinese-cultivated *P. cablin* is divided into four cultivars and two chemotypes. Traditionally, PA-type cultivars have mainly been used in the perfume industry, whereas PO-type cultivars have been considered medicinal plants in China (Zhang et al., 2019a). Since the traits of different cultivars (chemotypes) are very similar, it is morphologically difficult to distinguish the two chemotypes in the commercial market. Therefore, several problems, such as confusion regarding the source of the cultivars and difficulty in distinguishing the chemotypes, have appeared. Furthermore, the yield and quality of *P. cablin* cultivars in China are extremely unstable, which has become a serious problem restricting the development of the patchouli industry (Xu et al., 2015). The method of molecular marker-assisted breeding has been widely used in practical efforts aimed at germplasm improvement in field crops. Breeding *P. cablin* cultivars with stable yields and high contents of medicinal ingredients is of great significance for *P. cablin* production. SSRs are very popular codominant markers that have been widely employed in germplasm identification, genetic breeding, gene mapping, and genetic diversity and genetic structure analyses because of their high polymorphism, high variation, high sensitivity, codominant inheritance, stability and reliability (Zhang et al., 2021). For the identification of SSR markers, transcriptome sequencing is the recommended approach that is adopted in many studies (Zhou et al., 2016). Diverse types of SSRs obtained from RNA-Seq have been widely used in genetic diversity analyses in plants. To identify SSRs, we searched all unigenes of the two *P. cablin* cultivars with MISA software. A total of 8,314 SSRs were identified in 6825 unigenes, with a distribution frequency of 18.32%, among which 1,202 unigenes contained more than one SSR. There was a large proportion of mono-, di-and trinucleotide motifs (96.5%), while the other types accounted for < 2% of the repeat motifs. This is consistent with the SSR distributions reported in cotton (Hamid et al., 2018), *Aconitum carmichaelii* (Rai et al., 2017), and *Chrysanthemum* × *grandiflorum* (Ramat.) Kitam (Dong et al., 2018). The percentage of SSR-containing sequences was higher than those in *Areca catechu* L (Manimekalaia et al., 2018) and *Dipteronia* Oliver (Zhou et al., 2016). These markers obtained from the transcriptome have the potential to be used in quantitative trait locus (QTL) mapping, genetic diversity studies, and the fingerprinting of cultivars in *P. cablin*.

## Conclusion

In this study, we first report the transcriptome information of Chinese-cultivated *P. cablin* cv. pogostone-type “Paixiang” and patchoulol-type “Nanxiang” and provide comprehensive information on the biosynthetic pathway and molecular regulation of patchouli alcohol. The two chemotypes generated 36.83 G clean bases; these were compared with seven databases and revealed 45,394 annotated unigenes. A total of 8390 DEGs were identified between the cultivars, including 2,467 upregulated and 5,923 downregulated unigenes in PX. There were 36 candidate unigenes involved in PA biosynthesis. One key sesquiterpene synthase gene involved in the sesquiterpenoid and triterpenoid biosynthetic pathways encodes patchoulol synthase variant 1, which was significantly upregulated in NX. Overall, 23 AP2/ERF, 13 bHLH, 11 MYB, 11 NAC, three Trihelix, 10 WRKY and three bZIP genes were differentially expressed and therefore may act as regulators in the biosynthesis of PA and other terpenoids. Moreover, a total of 8,314 SSRs in 6,825 unigenes were identified. This study will help to elucidate the biosynthetic pathways and molecular regulation mechanism of PA and will contribute to molecular plant breeding. However, the specific mechanisms involved still need to be further studied.

## Supplemental Information

10.7717/peerj.12025/supp-1 Supplemental Information 1 The sequences of the selected genes of qRT-PCR.

10.7717/peerj.12025/supp-2 Supplemental Information 2 Unigene distribution of KOG functional classification.

10.7717/peerj.12025/supp-3 Supplemental Information 3 Number of unigenes annotated with GO terms.

10.7717/peerj.12025/supp-4 Supplemental Information 4 KEGG pathway classification of assembled unigenes.

10.7717/peerj.12025/supp-5 Supplemental Information 5 Differentially expressed genes between PX and NX.

10.7717/peerj.12025/supp-6 Supplemental Information 6 KEGG pathway annotation of assembled unigenes.

10.7717/peerj.12025/supp-7 Supplemental Information 7 Enriched KEGG pathways of DEGs.

10.7717/peerj.12025/supp-8 Supplemental Information 8 Identification of transcription factors between PX and NX.

10.7717/peerj.12025/supp-9 Supplemental Information 9 Details of SSR repeats.

10.7717/peerj.12025/supp-10 Supplemental Information 10 Possible SSR specific primers.

10.7717/peerj.12025/supp-11 Supplemental Information 11 The raw data of qRT-PCR assay.

10.7717/peerj.12025/supp-12 Supplemental Information 12 Primer sequences for qRT-PCR.

10.7717/peerj.12025/supp-13 Supplemental Information 13 Statistical analyses and mapping results of RNA sequencing reads to unigene.

10.7717/peerj.12025/supp-14 Supplemental Information 14 The length distribution (a) and GC contents (b) of assembled unigenes.

10.7717/peerj.12025/supp-15 Supplemental Information 15 Terpenoid backbone biosynthetic pathway. Blue represents no differentially expressed unigenes, red represents upregulated unigenes, green represents downregulated unigenes, and yellow represents both upregulated and downregulated unigenes. Blue represents no differentially expressed unigenes, red represents upregulated unigenes, green represents downregulated unigenes, and yellow represents both upregulated and downregulated unigenes.

10.7717/peerj.12025/supp-16 Supplemental Information 16 Sesquiterpenoid and triterpenoid biosynthetic pathway. Blue represents no differentially expressed unigenes, red represents upregulated unigenes, green represents downregulated unigenes, and yellow represents both upregulated and downregulated unigenes. Blue represents no differentially expressed unigenes, red represents upregulated unigenes, green represents downregulated unigenes, and yellow represents both upregulated and downregulated unigenes.

10.7717/peerj.12025/supp-17 Supplemental Information 17 The assembled unigenes.

## Additional Information and Declarations

### Data Availability

The following information was supplied regarding data availability: Data are available at the NCBI Sequence Read Archive: PRJNA660501 and PRJNA660544. The transcriptome shotgun assembly project is available at DDBJ/EMBL/GenBank: GJHT00000000. The version described in this paper is the first version, GJHT01000000.
