# Supplementary material for: Transcriptome analysis of two Pogostemon cablin chemotypes reveals genes related to patchouli alcohol biosynthesis
Source: PeerJ. 2021 Aug 26;9:e12025. doi: 10.7717/peerj.12025 (PMC8403477; doi:10.7717/peerj.12025)
Supplement: Supplemental Information 12 [file peerj-09-12025-s012.docx]

| **unigene** | **Primer sequences** |
| --- | --- |
| TRINITY_DN15930_c0_g1_i5 | F:GCTGGCAACAAAGACC |
|  | R:TCAAACTCGCATCCTG |
| TRINITY_DN20235_c0_g1_i3 | F:GAGGGACCAGATGACAATAAC |
|  | R:ACTCCAAGCACCGAAAGATA |
| TRINITY_DN24538_c2_g3_i1 | F:ACCTACTTCTGGGATTGTGG |
|  | R:CAAAGAGTCGCAGTTCGTCA |
| TRINITY_DN27775_c0_g2_i2 | F:GAATACTGGCTTCTTCCCTC |
|  | R:TGATAGGACCCACAAACTTCT |
| TRINITY_DN30536_c2_g1_i3 | F:GAGGACCCTAATGGAGACTAT |
|  | R:CCGTATCCCACTCTTGACTT |
| TRINITY_DN18757_c0_g1_i1 | F:ATGGCGATGGCGTCCAATAC |
|  | R:ACGGCGGAAATAGAGGAGCA |
| TRINITY_DN22349_c0_g2_i1 | F:TTTTATTCGCTATTGGAAGACG |
|  | R:ATCTACACCGACTACGATACCAA |
| TRINITY_DN21681_c0_g1_i2 | F:CTGGACAACGGGTCGTATTA |
|  | R:TACAGTCCTTGCTGGTGATG |
| TRINITY_DN14252_c0_g1_i1 | F:CGGCTGTCATAAACGA |
|  | R:CCACCCTACTTCCCTCT |
| TRINITY_DN14955_c0_g1_i1 | F:CAGTTTCTTGCCATTGCTTC |
|  | R:CGTCGCTAGGATTACTACCAT |
| TRINITY_DN29365_c0_g2_i1 | F:CTGGACTTGGTATGGCTGTG |
|  | R:CTTCGTATGCTTGTCCTGCT |
| TRINITY_DN30344_c0_g2_i7 | F:ATCAGGTGGTTCACGATGTAG |
|  | R:CAGTGGAGTTCCCTTGTTGT |
| 18S rRNA | F:CCGACCATAAACGATGCCGACC |
|  | R:TTTCAGCTTTGCAACCATACTCC |
