# Supplementary material for: Transcriptome analysis of two Pogostemon cablin chemotypes reveals genes related to patchouli alcohol biosynthesis
Source: PeerJ. 2021 Aug 26;9:e12025. doi: 10.7717/peerj.12025 (PMC8403477; doi:10.7717/peerj.12025)
Supplement: Supplemental Information 13 [file peerj-09-12025-s013.docx]

| **Term\Sample** | **PX1** | **PX2** | **PX3** | **NX1** | **NX2** | **NX3** |
| --- | --- | --- | --- | --- | --- | --- |
| Total reads | 43142174(100.00%) | 43540052(100.00%) | 39681932(100.00%) | 40710992(100.00%) | 41888242(100.00%) | 53282330(100.00%) |
| Total mapped reads | 39365799(91.25%) | 39701128(91.18%) | 36068793(90.89%) | 36084106(88.63%) | 37116560(88.61%) | 47453856(89.06%) |
| Multiple mapped | 1767951(4.10%) | 1762370(4.05%) | 1567478(3.95%) | 1786980(4.39%) | 1831451(4.37%) | 2304010(4.32%) |
| Uniquely mapped | 37597848(87.15%) | 37938758(87.14%) | 34501315(86.94%) | 34297126(84.25%) | 35285109(84.24%) | 45149846(84.74%) |
| Reads mapped in proper pairs | 36790170(85.28%) | 37149756(85.32%) | 33752790(85.06%) | 33511922(82.32%) | 34491868(82.34%) | 44153420(82.87%) |
