# Supplementary figures and images for: Transcriptome analysis of two Pogostemon cablin chemotypes reveals genes related to patchouli alcohol biosynthesis
Source: PeerJ. 2021 Aug 26;9:e12025. doi: 10.7717/peerj.12025 (PMC8403477; doi:10.7717/peerj.12025)

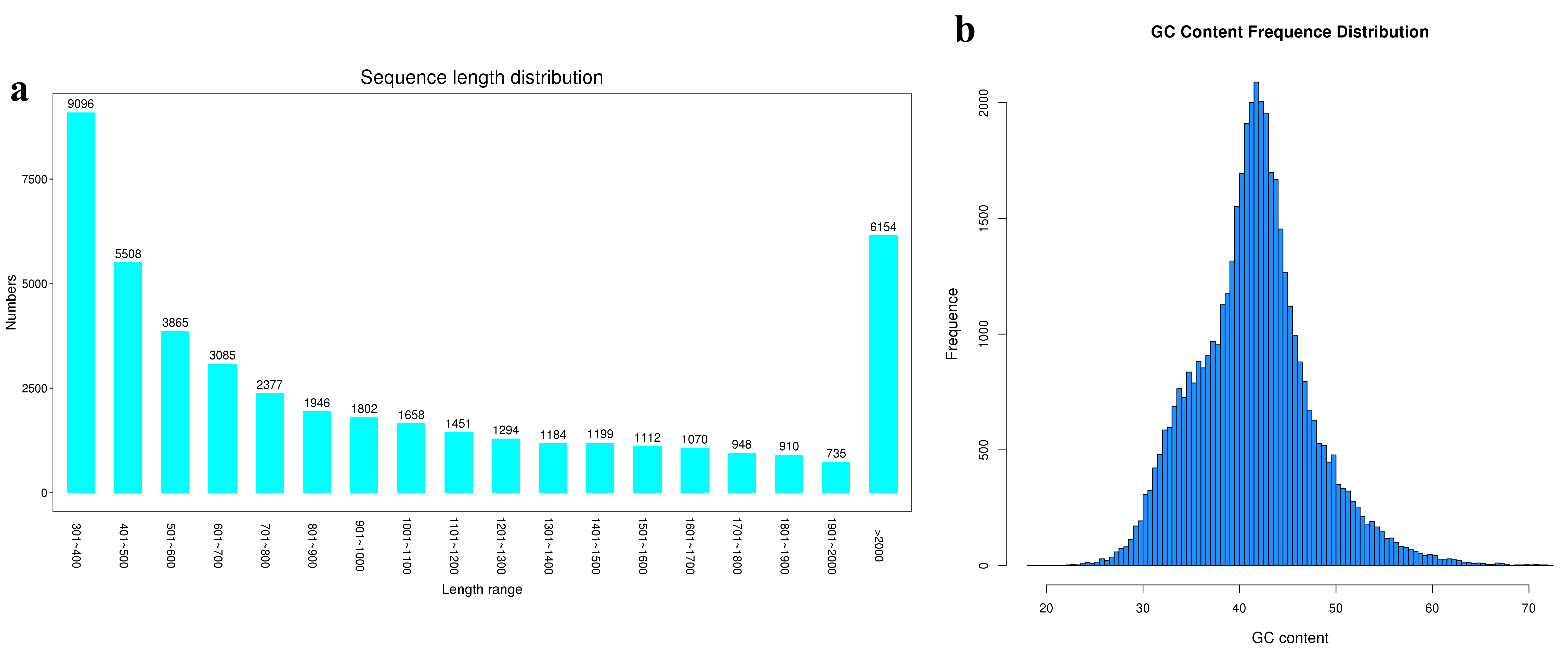

Supplement: Supplemental Information 14 [file peerj-09-12025-s014.png]

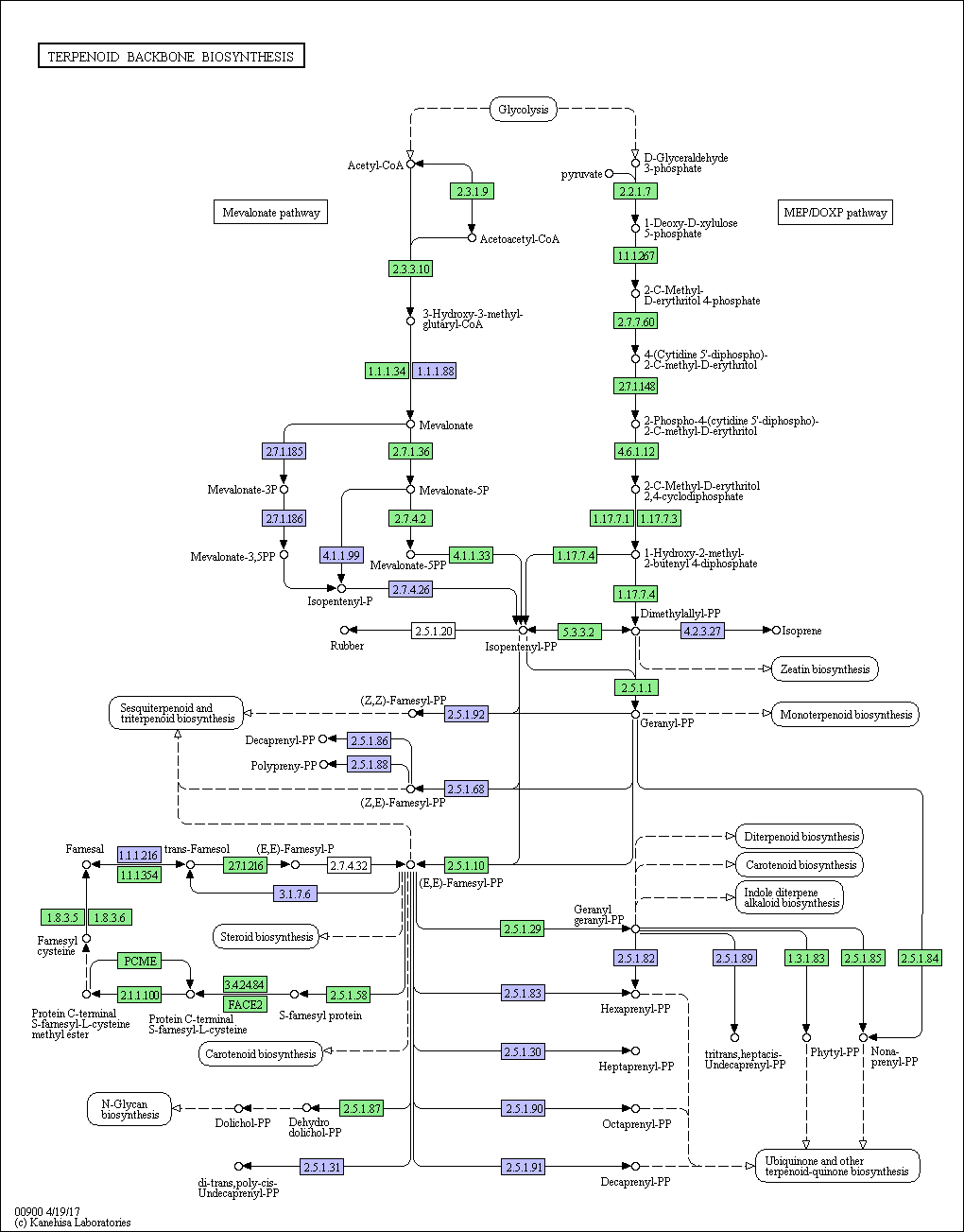

Supplement: Supplemental Information 15 — Blue represents no differentially expressed unigenes, red represents upregulated unigenes, green represents downregulated unigenes, and yellow represents both upregulated and downregulated unigenes. [file peerj-09-12025-s015.png]

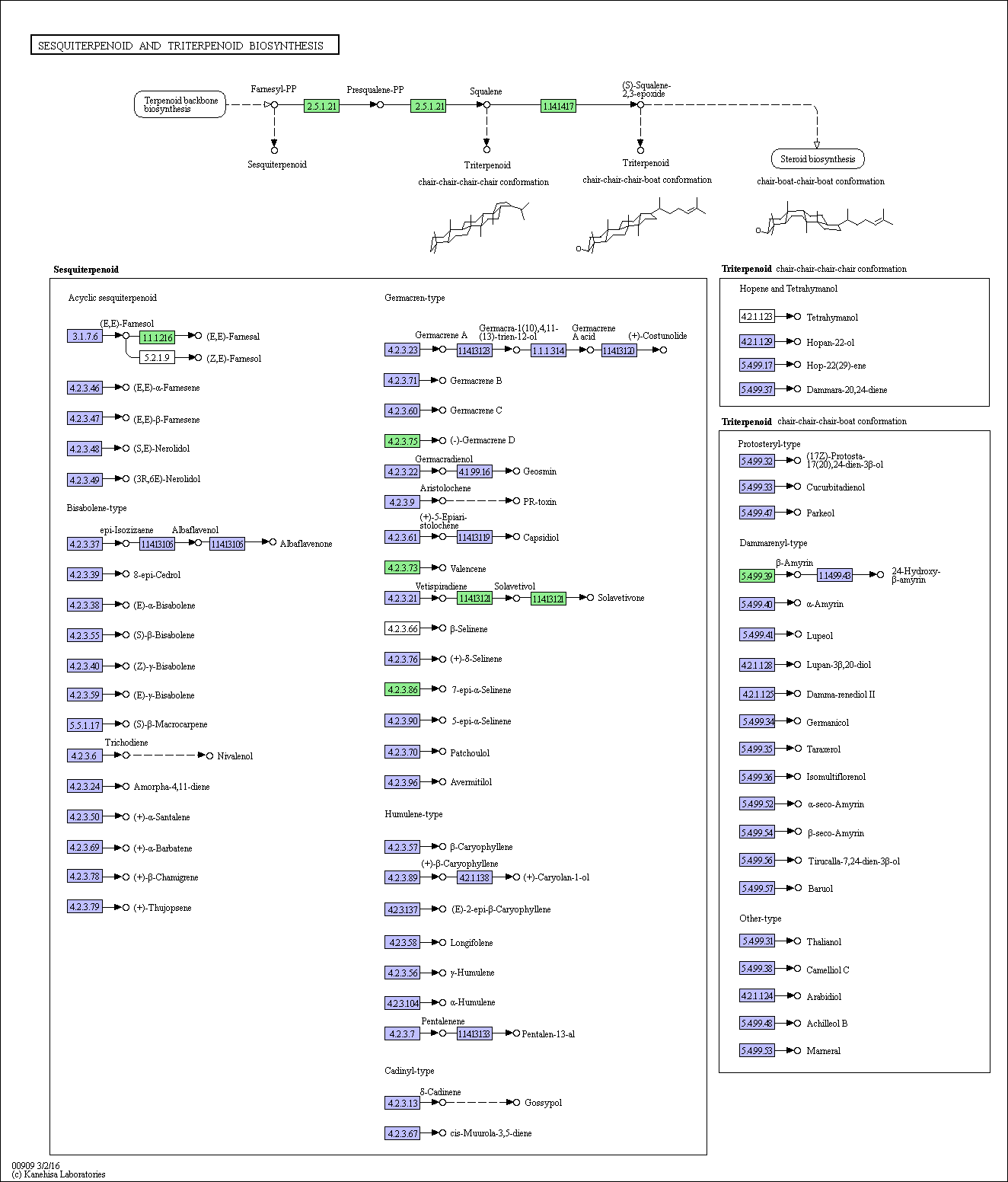

Supplement: Supplemental Information 16 — Blue represents no differentially expressed unigenes, red represents upregulated unigenes, green represents downregulated unigenes, and yellow represents both upregulated and downregulated unigenes. [file peerj-09-12025-s016.png]
